# A holistic view of mouse enhancer architectures reveals analogous pleiotropic effects and correlation with human disease

**DOI:** 10.1186/s12864-020-07109-5

**Published:** 2020-11-02

**Authors:** Siddharth Sethi, Ilya E. Vorontsov, Ivan V. Kulakovskiy, Simon Greenaway, John Williams, Vsevolod J. Makeev, Steve D. M. Brown, Michelle M. Simon, Ann-Marie Mallon

**Affiliations:** 1 Mammalian Genetics Unit, MRC Harwell Institute, Oxfordshire, OX11 0RD UK; 2 Vavilov Institute of General Genetics, Russian Academy of Sciences, Gubkina 3, Moscow, 119991 Russia; 3 Institute of Protein Research, Russian Academy of Sciences, Institutskaya 4, Pushchino, Moscow Region 142290 Russia; 4 Engelhardt Institute of Molecular Biology, Russian Academy of Sciences, Vavilova 32, Moscow, 119991 Russia; 5 Institute of Translational Medicine, University Hospitals Birmingham NHS Foundation Trust, Birmingham, B15 2TH UK; 6 Institute of Cancer and Genomic Sciences, University of Birmingham, Birmingham, B15 2TT UK; 7 Moscow Institute of Physics and Technology, 9 Institutskiy per., Dolgoprudny, Moscow Region 141700 Russia

**Keywords:** Super-enhancers, Typical-enhancers, Tissue-specificity, Expression, Phenotypes, Protein-protein interactions, Transcription factors, Gene-phenotype prediction

## Abstract

**Background:**

Efforts to elucidate the function of enhancers in vivo are underway but their vast numbers alongside differing enhancer architectures make it difficult to determine their impact on gene activity. By systematically annotating multiple mouse tissues with super- and typical-enhancers, we have explored their relationship with gene function and phenotype.

**Results:**

Though super-enhancers drive high total- and tissue-specific expression of their associated genes, we find that typical-enhancers also contribute heavily to the tissue-specific expression landscape on account of their large numbers in the genome. Unexpectedly, we demonstrate that both enhancer types are preferentially associated with relevant ‘tissue-type’ phenotypes and exhibit no difference in phenotype effect size or pleiotropy. Modelling regulatory data alongside molecular data, we built a predictive model to infer gene-phenotype associations and use this model to predict potentially novel disease-associated genes.

**Conclusion:**

Overall our findings reveal that differing enhancer architectures have a similar impact on mammalian phenotypes whilst harbouring differing cellular and expression effects. Together, our results systematically characterise enhancers with predicted phenotypic traits endorsing the role for both types of enhancers in human disease and disorders.

## Background

Mammalian gene expression and their parallel gene networks are tightly controlled by non-coding regulatory regions such as enhancers, their accompanying transcription factors (TFs), chromatin re-modellers and non-coding RNAs [1]. Large scale programs such as ENCODE [2], FANTOM5 [3] and NIH Roadmap Epigenomics project [4] have generated an initial detailed exploration of active enhancer and promoter regions in a plethora of tissues and cell types forming a crucial data source for study of regulatory regions. Putative enhancers have been predicted in multiple organisms with > 1 million estimated in the mouse and human genomes [2, 5–8]. ChIP-Seq analysis of chromatin modification has been widely used to catalogue these potential enhancer and promoter regions, with enhancer loci being enriched in histone H3 lysine4 monomethylation (H3K4me1) and lacking histone H3 lysine4 trimethylation (H3K4me3), while active enhancer sites have the addition of histone H3 lysine27 acetylation (H3K27ac) [5, 9]. Contrastingly, active promoter regions have an enrichment of H3K4me3 and H3K27ac, and a depletion of H3K4me1 [5, 10]. Although these elements have been comprehensively identified, catalogued and archived, numerous questions still remain on the interpretation of their biological relevance, effect on gene expression, and overall impact on disease causation.

Stringent control of transcription is required for the correct functioning of multicellular organisms, with different regulatory regions occupying different roles; promoters initiate transcription while enhancers control the correct spatio-temporal expression of genes [11]. Looping of the chromatin brings the enhancers close to the promoter regions of their target genes [12–14]. As a result, the enhancers increase the rate of transcription by increasing the number of factors involved in the process. Most important factors among these include the Mediator complex, which is a co-activator complex binding to other TFs and RNA polymerase II [15]; cohesin, which stabilises and sometimes even drives cell-type specific enhancer-promoter communication bridges [15]; and factors important for paused RNA polymerase II release and elongation such as *BRD4* [16]. How these interactions and chromatin looping are established remains largely unknown. However, regulatory elements; TFs, chromatin modellers, enhancers and promoters must be in close concert to promote transcription, while their disruption may lead to disease in humans and related phenotypes in model organisms such as mouse [11, 17, 18]**.** Furthermore, over 90% of GWAS SNPs associated with human disorders occur within the non-coding regions, with 64% of the non-coding SNPs in enhancer (H3K27ac positive) regions [19–21]. Similarly, ~ 76% of non-coding SNPs from GWAS are identified either within DNaseI hypersensitive sites (DHS) or in high linkage disequilibrium with a SNP within DHS [20]. Indeed, the number and scale of putative disease variants identified in the non-coding genome has driven the characterisation of enhancers and their association to pathological states. The pathology of disease in humans is commonly studied in the laboratory mouse, typically by analysing the phenotypes arising from targeted mutations. Phenotyping initiatives like the International Mouse Phenotyping Consortium (IMPC) [22, 23] identify phenotype-genotype associations by producing mouse lines with a protein-coding gene knockout and systematically recording the results from a battery of phenotyping tests for each line. These standardised tests cover a multitude of biological processes and provide consistent descriptions of phenotypes for each functional gene, which can be used in the understanding of human traits and diseases. As with the coding regions of the mouse genome, the study of enhancers and other non-coding regions has been greatly facilitated by CRISPR and on a case-by-case basis we are beginning to understand the roles of enhancers in the susceptibility and pathogenesis of disease [24–30]. However, despite recent progress in the study of the non-coding genome, systematic genotype-phenotype analysis of enhancers and other non-coding regions remains a substantial challenge.

Recently, dense clusters of active enhancers have been recognised as a new class of regulatory element termed super-enhancers (SEs) [31]. These elements spanning large genomic regions are enriched with various chromatin regulators and cofactors such as the Mediator complex, p300, Brd4 and RNA polymerase II [21]. Mediator binding and H3K27ac chromatin marks have been most commonly used to segregate SEs from regular enhancers referred to as typical-enhancers (TEs). Systematic mapping of SEs using H3K27ac chromatin mark across diverse human tissues and cell lines show that SEs regulate genes that define cell identity and drive high expression of their target genes compared to TEs [21, 32–34]. While studies in the mouse genome find similar results, they are currently limited to relatively few tissue types [31, 35–39]. Furthermore, SEs in human cell types have been shown to frequently harbour disease-causing variation [21, 40, 41], while TEs have been considered less important. However, to date there has been no systematic study defining genome-wide functional difference between SEs and TEs, and their relationship to phenotypes.

Here, we systematically identified highly tissue-specific enhancers in 22 mouse tissues, and further classified them into SEs and TEs. Moreover, we linked these enhancers with genes associated with phenotypic effects in the mouse. We find that though SEs drive high total-expression (aggregated expression of all exons) and tissue-specific expression (tendency of gene to be specifically expressed in a tissue or cell line) of their associated genes, large number of TEs in the genome enable them to contribute greatly to the tissue-specific expression landscape. For the first time our results show both SE and TE associated genes are enriched for relevant phenotypes and diseases in the corresponding tissue-types, and we show there is no significant difference in severity and breadth of phenotypes produced from knockouts of SE and TE associated genes, indicating the importance of both enhancer types in disease causation. We go on to use regulatory data combined with other molecular characteristics to infer mammalian gene-phenotype associations and identify potential novel pathogenic genes which may be used for further characterisation.

## Results

### Systematic profiling of tissue-specific regulatory elements (TSREs) in mouse

**Fig. 1:**
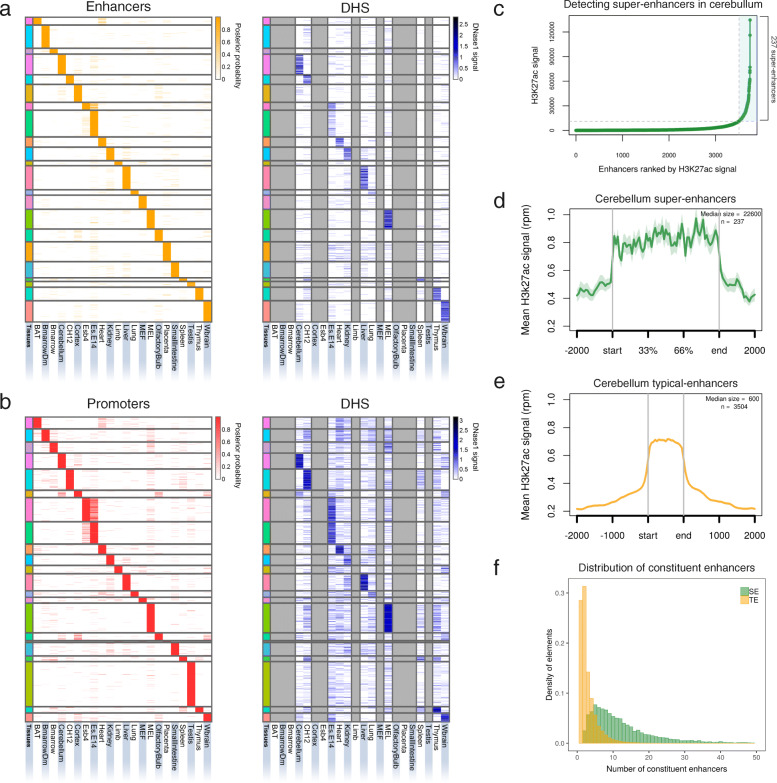
Overview of TSREs identified in 22 mouse tissues. **a** Strong enhancers, **b** Active promoters: Heatmaps showing chromatin state posterior probability of tissue-specific regulatory elements (Tau_reg_ ≥ 0.85) (left) and their corresponding DNAse1 signal (right) in every tissue. Each row is a genomic location and columns represent different mouse tissues and cell lines. Grey columns show tissues for which data was not available. The heatmaps have been sorted by the order of the tissues across the columns. (BAT: Brown Adipose Tissue; Bmarrrow: Bone Marrow; BmarrowDm: Bone Marrow derived macrophage; CH12: B-cell lymphoma; Esb4: mouse embryonic stem cells; Es-E14: mouse embryonic stem cell line embryonic day 14.5; MEF: Mouse Embryonic Fibroblast; MEL: Leukaemia; Wbrain: Whole Brain). **c** Distribution of H3K27ac ChIP-seq signal over cerebellum-specific enhancers stitched together within 12.5 kb (*n* = 3741). Stitched cohesive units (x-axis) are ranked in an increasing order of their input-normalised H3K27ac signal (reads per million, y-axis). This approach identified 237 SEs (highlighted in blue) and 3504 TEs in cerebellum. **d**-**e** Metagene profile of mean H3k27ac ChIP-seq signal across all the SEs and TEs in cerebellum. The profiles are centred on the enhancer regions and the surrounding 2 kb regions around each enhancer is shown. The length of the enhancer region is scaled to represent the median size of SEs (22,600 bp) and TEs (600 bp) in cerebellum. The shaded area shows the standard error (SEM). **f** Distribution of constituent enhancers within SEs and TEs across all 22 tissues. See also Additional file 1: Figure S2-S5

To systematically identify potential regulatory elements in the mouse genome, we annotated genome-wide chromatin states using a multivariate hidden Markov model called ChromHMM [42]. We constructed the model using three primary histone marks (namely H3K4me1, H3K4me3 and H3K27ac) in 22 mouse epigenomes from ENCODE [2]. These chromatin states can be broadly categorised into active promoter, weak promoter, strong enhancer and weak enhancer states (Additional file 1: Figure S1). Overall, we annotated 923,791 strong enhancer and 309,581 active promoter annotations (each being 200 bp in length) across the 22 epigenomes (posterior probability of states ≥0.95). To validate the accuracy of our predicted promoters and strong enhancers, we compared them to known promoter and enhancer elements in the mouse genome (see methods). The predicted regulatory elements achieved a recall sensitivity of 81.7% (18,543/22,707) for the promoters of protein-coding genes, and 91.2% (331/363) for enhancers. To accurately identify mouse TSREs, we implemented the previously described TAU algorithm [43, 44] to calculate the tissue specificity index (*τ*_*reg*_) of every strong enhancer and active promoter (see methods). In total across 22 mouse tissues, 31% of all strong enhancers were shown to be highly tissue-specific (*τ*_*reg*_ ≥ 0.85) and 43% of active promoters. Both, also show a high degree of positive correlation with DNaseI hypersensitive sites (DHS) in the corresponding tissues (Pearson’s correlation, *p* < 2.2e-16), confirming these TSREs are highly tissue-specific (Fig. 1a-b, Additional file 1: Figure S2).

To identify mouse SEs, we used the ROSE algorithm [31] to combine tissue-specific enhancer elements within a span of 12.5 kb into cohesive units and rank them based on H3K27ac signal which distinguishes them from TEs (Fig. 1c). The enhancer elements within the cohesive units (for both categorised as SEs or TEs) are referred to as constituent enhancers (Additional file 1: Figure S2d). Using this approach, 6.6% (5082) of all cohesive units (or 24% of all tissue-specific enhancers) are SEs while 93.4% (71,824) are TEs (or 76% of all tissue-specific enhancers) (Additional file 1: Figure S2e). As expected, we found SE cohesive units are occupied on average by 2.4x H3K27ac and span large genomic regions (median size = 12.4 kb) compared to TEs (median size = 0.4 kb) (Fig. 1d-e, Additional file 1: Figure S3). The number of constituent enhancers are enriched in SEs compared to TEs (Fig. 1f). Enrichment of H3K4me1 and DHS at SEs is observed to be in agreement with H3K27ac levels (Additional file 1: Figure S4). To determine whether the high levels of histone modification activity at SEs are a consequence of the total genomic length of their cohesive units, we compared the enrichment of H3K27ac and H3K4me1 among their constituent enhancers to TEs. We find that constituent enhancers within SEs show a higher density of H3K27ac and H3K4me1 histone marks compared to TEs (Additional file 1: Figure S5a and S5b), suggesting the increased levels of chromatin activity in SEs is not a consequence of the total genomic length of their cohesive units. A similar trend was identified for RNA polymerase II indicating a potential role of enhancer RNAs (eRNAs) in enhancer activity and gene regulation, as reported in recent studies [45, 46] (Additional file 1: Figure S5c).

SEs have been found to frequently overlap the genes they regulate [21, 31]. A previous study in murine ESCs identified more than 80% of SEs and TEs to interact with their nearest active gene [47]. To explore the functional role of enhancers we associated each enhancer element to a potential target gene using a community accepted tool, GREAT [48]. We identified 3617 and 14,791 protein-coding genes associated with SEs and TEs in at least one tissue or cell type, respectively (Additional file 2). The resulting enhancer-gene associations were highly consistent with previously identified topological associated domains (TADs) (96% in cortex TADs and 93% in mESC TADs) [49] (Additional file 1: Figure S6a, Additional file 3). Similarly, 87% of associations overlapped with computationally derived enhancer-promoter units (EPUs) [6]. As expected, the majority (62.53% of SEs, 57.25% of TEs) of the tissue-specific enhancers are located within 50 kb from the transcription start sites (TSSs) of their associated genes (Additional file 1: Figure S6b-S6d). The predicted SEs, TEs and their associated genes were used for all subsequent analysis.

### Typical and super-enhancers can boost tissue-specific gene expression

Previous studies in human and mouse cell types have shown SEs to be related with highly expressed genes [21], however the studies in mouse were less comprehensive and limited to a few tissues [31, 35, 39, 50]. In addition to this total-expression, a few studies have demonstrated SEs to be associated with tissue-specific gene expression in cell lines. For instance, genes associated with SEs in multiple myeloma cell lines were preferentially expressed in myeloma cells [32]. With the aim of exploring whether this association prevails genome-wide, across multiple tissue types and different enhancers, we examined the impact of these newly identified enhancers in 22 tissues. To inspect this, we utilised ENCODE RNA-Seq data. To effectively identify any common expression patterns between genes, tissues and enhancers, we constructed a dataset formed of genes expressed within a particular tissue, termed gene-tissue pairs, followed by categorisation on their type of enhancer association, hence grouping them into three classes: (1) gene-tissue pairs associated with SEs, referred to as super-enhancer class (SEC); (2) gene-tissue pairs associated with TEs, referred to as typical-enhancer class (TEC); and (3) gene-tissue pairs associated with weak/poised enhancers, referred to as weak-enhancer class (WEC).

**Fig. 2:**
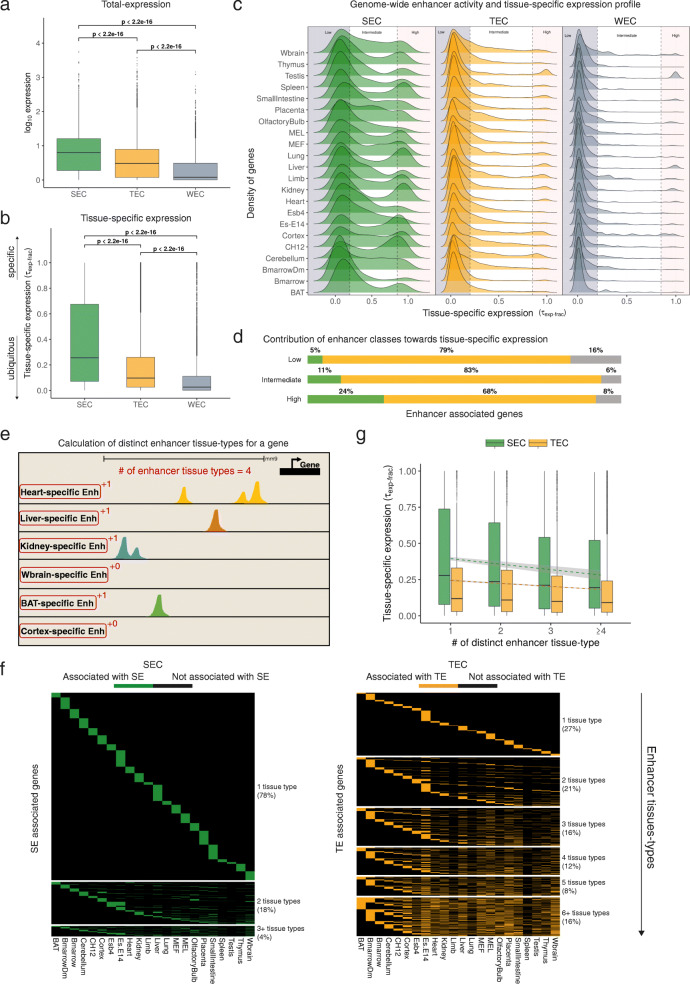
SEs promote high transcriptional activity and drive tissue-specific expression in mouse. **a** Box plot showing the total-expression (in log-transformed RPKM) of different enhancer classes across 22 tissues. Each box plot shows the median, middle bar; interquartile range, the box; whiskers, 1.5 times the interquartile range. **b** Box plot showing the tissue-specific expression of different enhancer classes across 22 tissues. The *p*-values were calculated using Wilcoxon Rank Sum Test. **c** Distribution of genes within tissue-specific expression categories (low, intermediate and high) in different enhancer classes. Y-axis for each tissue displays the density of genes scaled across the tissues, but not across the enhancer classes. **d** Contribution of each enhancer class (in percentage) towards the total number of enhancer associated genes in the genome, categorised by their tissue-specific expression. **e** A schematic to illustrate the calculation of distinct enhancer tissue-types for each enhancer-associated gene. The number of distinct tissue types of various enhancers associated with the gene of interest are added to compute the number of enhancer tissue-types for a gene. **f** Heatmaps showing the number of enhancer tissue-types in SEC and TEC. Each row is an enhancer associated gene and columns represent its association with enhancers across 22 tissues and cell types. **g** Box plot showing the correlation between the number of enhancer tissue-types and tissue-specific expression of SEC and TEC. The trend lines (green: SEs; orange: TEs) were calculated using linear regression. See also Additional file 1: Figure S7 and S8

We found that both SEC and TEC are associated with highly expressed genes in comparison to the WEC (SEC: effect size (ES) = 0.95, *p* < 2.2 × 10^− 16^; TEC: ES = 0.86, *p* < 2.2 × 10^− 16^; Wilcoxon Rank Sum Test) but that the SEC appears to have the highest level of total-expression (SEC compared to TEC: ES = 0.56, *p* < 2.2 × 10^− 16^) (Fig. 2a, Additional file 1: Figure S7a). Likewise, the SEC have higher tissue-specific expression (quantified as *τ*_*exp* − *frac*_, see methods) compared to the TEC (ES = 0.62, *p* < 2.2 × 10^− 16^; Wilcoxon Rank Sum Test) or WEC (ES = 0.96, *p* < 2.2 × 10^− 16^) (Fig. 2b). To further understand tissue-specific expression of the genes within different enhancer classes, we categorised it into three levels of low, intermediate and high (see methods). We identified, 16.46% (690/4191) of SEC, 4.42% (1923/43,484) of TEC and 3.38% (230/6795) of WEC to have high tissue-specific expression (Fig. 2c, Additional file 1: Figure S7b). Further examination of the high tissue-specific expression category shows the absolute number of genes within the TEC (1923) is notably higher than in the SEC (690) or WEC (230). Overall this data suggests the ratio of genes within the SEC with high tissue-specific expression is at least 4 times larger than the genes within other enhancer classes. However, their absolute number is smaller compared to the TEC which contribute the largest amount (68%) of enhancer associated tissue-specific expression in the genome (Fig. 2d). This body of work in mouse strengthens the theory that super-enhancers can boost tissue-specific gene expression, while highlighting that high numbers of typical-enhancers, can also boost tissue-specific expression and should not be overlooked.

While identifying SEs we observed they are comprised of a large number of constituent enhancers (Fig. 1f). The average number of constituent enhancers within SEs is 13, compared to 3 in TEs. To this end, we examined whether an increase in the number of constituent enhancers results in an increase in total-expression of their associated genes. To increase the power of this analysis, we combined both the SEC and TEC into a single dataset. We correlated the frequency of the constituent enhancers (total number of constituent enhancers associated with a gene) within the combined dataset with total-expression of their associated gene, which revealed a weak positive correlation (Spearman’s correlation *rho* = 0.12, *p* < 2.2 × 10^− 16^) (Additional file 1: Figure S8a). To ensure this observation was not driven predominantly by one class of enhancer, we examined this correlation separately within SEC and TEC, and found no notable difference between the two classes (Additional file 1: Figure S8b and S8c). In contrast, weak-enhancer elements show little to no correlation with total-expression (Spearman’s correlation *rho* = − 0.03, *p* = 0.02) of their associated genes (Additional file 1: Figure S8d). Overall this shows that total-expression of a gene modestly increases with an increase in the number of constituent enhancers, indicating a non-additive relationship between them. This suggests that constituent enhancers appear to exert a complex, instead of a simple additive effect on the transcriptional output.

Since a gene could be related to SEs or TEs in multiple tissues, we inspected these multiple gene-enhancer associations for their effect on tissue-specific expression. For this purpose, we assessed the number of distinct tissues, where an enhancer associated with a gene occurs, which we define here as “enhancer tissue-types” (Fig. 2e). A large portion (∼78%, 2821 out of 3617) of the SEC is associated with one enhancer tissue-type, i.e. the genes are associated with SEs from one tissue (Fig. 2f). However, only 27% (3956 out of 14,791) of the TEC have one enhancer tissue-type, while the remaining 73% are associated with TEs of two or more tissues (Additional file 4 provides the list of these genes). Furthermore, we see that genes with a higher number of enhancer tissue-types are associated with low values of *τ*_*exp* − *frac*_ (Fig. 2g), hence increasing enhancer tissue-type association increases ubiquitous expression.

We next turned our attention to the genes which are associated with more than one enhancer tissue-type. Since these genes are associated with enhancers in multiple tissues (two or more), we sought to examine what type of enhancer has a higher propensity to adopt an “enhancer usage switch”. We define “enhancer usage switch” as the phenomenon where the enhancer usage associated with a gene could differ across multiple tissues. We use the number of constituent enhancers (within SEs or TEs) associated with a gene-tissue pair as a measure of its enhancer usage. The standard deviation of its enhancer usage across the 22 tissues was used to predict the level of “enhancer usage switch”. A gene with a large “enhancer usage switch” score refers to an enhancer usage which varies highly across the different tissues. We compared the enhancer usage switch scores between SEC and TEC with multiple enhancer tissue-types, which shows that SEC exhibit significantly higher enhancer usage switch across the tissues (ES = 0.89, *p* < 2.2 × 10^− 16^; Wilcoxon Rank Sum Test) (Additional file 1: Figure S9). The genes with a high enhancer usage switch score for SEC include: *Ntm*, *Grm4*, *Foxa2*, and *Max*, whereas the genes with a high enhancer usage switch score for TEC include: *Csmd1, Ntrk3, Grin2a* and *Opcml* (Additional file 1: Figure S10; Additional file 5). Overall, this analysis shows that both SEC and TEC display enhancer usage switch, but SE usage of a gene varies significantly more across different cell- and tissue-types compared to TE.

### Enhancers drive phenotype and disease causation

Previous studies have identified SEs to be associated with genes that regulate cell identity and are therefore unlikely to be involved in a housekeeping role [21, 31]. To increase our understanding of the functional role of SE and TE associated genes we performed Gene Ontology (GO) enrichment analysis in 22 mouse tissues. Genes associated with SEs belonging to the SEC category are enriched for transcription factor binding activity (*p* = 10^− 10^), regulation of cell development (*p* = 10^− 16^) and regulation of cell differentiation (*p* = 10^− 23^) (Additional file 6). The breadth of this analysis demonstrates novel cell identity associations in unexplored tissues in the mouse. As expected, these are also important in the control and regulation of tissue or cell identity. Some examples of these novel SE associated genes include *Ucp1* (responsible for generating body heat in mammals [51]) in brown adipose tissue; *Gata4* (critical for heart development and cardiomyocyte regulation [52]) in heart; *Cxcr2* (regulates the emigration of neutrophils from bone marrow [53]) in bone marrow; and *Rbfox3* (splicing regulator of neuronal transcripts [54, 55]) in cerebellum. On the other hand, TEC appear to have different enrichments in GO analysis and are linked with genes involved in nucleotide and protein containing-complex binding (*p* = 10^− 6^), cellular protein localisation (*p* = 10^− 7^) and cell morphogenesis (*p* = 10^− 5^). Furthermore, TEC is significantly enriched for housekeeping genes (*p* = 2.7 × 10^− 11^, Odds Ratio (OR) = 1.49, 95% Confidence Intervals (CI) [1.32, 1.68]), while SEC is depleted (*p* = 0.012, OR = 0.82, 95% CI [0.69, 0.98]).

**Fig. 3:**
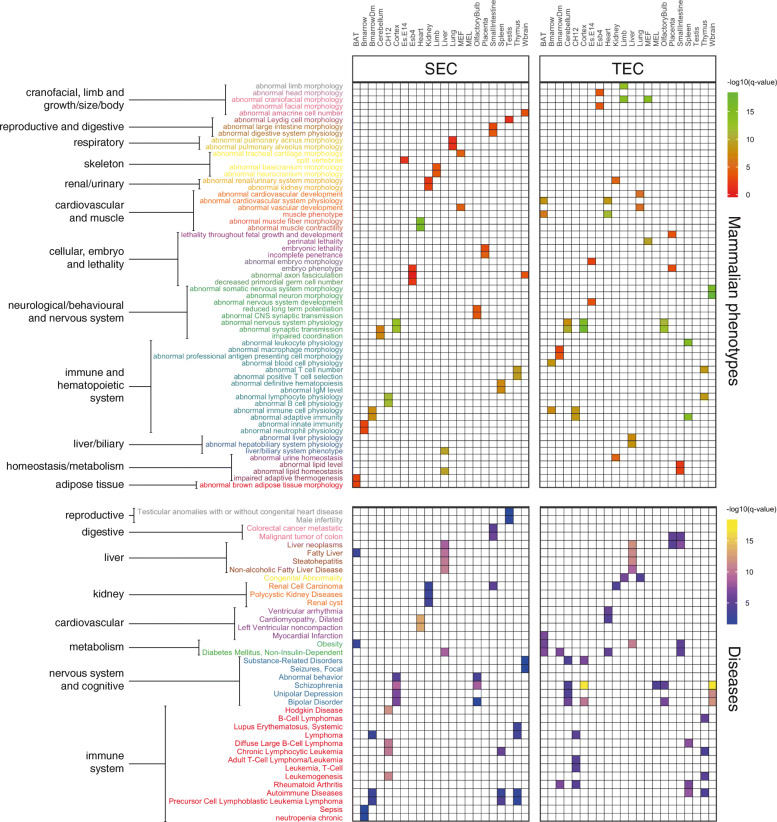
Mammalian phenotype and human disease ontology terms enriched in SEC and TEC. Listed are the most enriched mammalian phenotypes and human diseases among SEC and TEC in each tissue. The cells in the heatmap display the FDR (q-value) associated with the enriched terms and was calculated using the Benjamini-Hochberg method. The enrichment analysis was performed using ToppGene, which retrieves mouse phenotype annotations from MGD and human disease annotations from ClinVar, DisGenNet, GWAS and OMIM

To further explore the regulatory function of enhancers, we investigated mouse phenotypes and human diseases associated with genes within SEC and TEC (see methods). Significant enrichment in both phenotypes and disease ontology terms in the corresponding tissue types was identified (Fig. 3, Additional file 7), suggesting a strong relationship between both SEC and TEC and resulting pathological outcomes (disease causation). For instance, genes associated with cerebellum-specific enhancers are enriched for phenotypes such as impaired coordination (q = 4.83 × 10^− 8^) and abnormal synaptic transmission (q = 2.46 × 10^− 7^), and diseases such as bipolar disorder (q = 8.52 × 10^− 7^) and unipolar disorder (q = 6.26 × 10^− 5^). Similarly, genes related to heart-specific enhancers are enriched for phenotypes like abnormal cardiac muscle contractility (q = 9.05 × 10^− 16^) and diseases like cardiomyopathy (q = 5.45 × 10^− 14^) (Fig. 3). In addition, enrichment of blood-related cancers (such as Hodgkin Disease, q = 1.90 × 10^− 12^; T-cell Leukemia, q = 1.41 × 10^− 5^) in CH12 enhancer associated genes is consistent with the idea that oncogenes are placed under the effect of strong enhancers during cancer development leading to over-expression of these genes [32, 56]. On the other hand, the WEC display either an insignificant or a weak association with phenotypes in majority of the tissues (Additional file 1: Table S1).

**Fig. 4:**
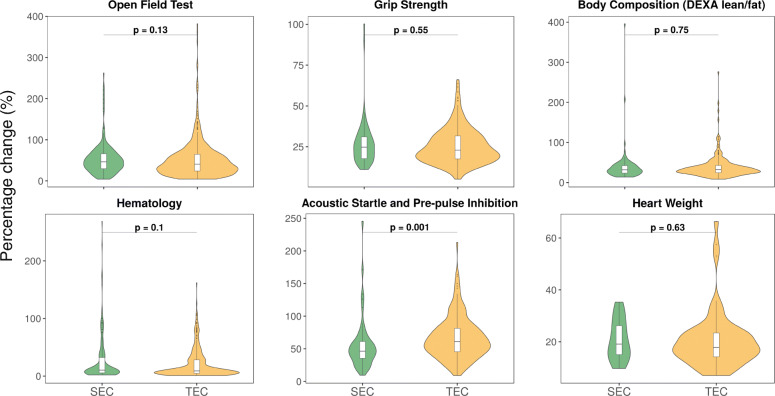
Phenotype severity of SE and TE associated gene knockouts. Violin plots showing the percentage change (normalised effect size) in phenotype procedures measured between enhancer associated gene knockouts and wild-type controls. The area under the violin is proportionate to the number of data points in each category. The *p*-values were calculated using the Wilcoxon Rank Sum Test. All phenotyping procedures show no significant difference in phenotype severity between SECs and TECs apart from Acoustic Startle and Pre-pulse Inhibition. See also Additional file 1: Figure S11 and S12

However, there is a marked difference in the expression patterns of SEC compared to TEC, which is not observed in their relationship with phenotypes. We explored this dichotomy further by comparing the phenotyping data from knockout mouse lines of genes in SEC and TEC across all tissues within the IMPC data. We reasoned that if SE associated genes are predominantly related to phenotype occurrence, their associated gene knockouts would cause a more severe phenotype condition (a phenotype with an increased effect size) relative to knockouts of other genes (such as those associated with TEs). We compared several standardised phenotyping procedures within the IMPC and observed a significant difference in severity only for acoustic startle and pre-pulse inhibition (ES = − 0.63, *p* = 0.001) (Fig. 4)**.** However, for the majority of the procedures, we observed no significant difference in severity of phenotypes between SEC and TEC (Open field test, ES = 0.19, *p* = 0.13; Grip strength, ES = 0.19, *p* = 0.55; DEXA, ES = − 0.02, *p* = 0.75; Heart weight, ES = 0.16, *p* = 0.63; Hematology, ES = 0.16, *p* = 0.1). Next, we sought to examine the breadth of the phenotypes associated with SEC and TEC. For this purpose, we computed the number of top-level phenotype ontology terms associated with SE and TE associated gene knockouts from IMPC (Additional file 1: Figure S11). No notable difference is observed in the breadth of phenotypes between SEC and TEC (ES = 0, *p* = 0.42), indicating both SE and TE associated gene knockouts are likely to produce comparable number of phenotypes and therefore, have similar pleiotropic effects. Furthermore, we explored the mouse essential genes by retrieving all the genes from IMPC which generate a lethal knockout [57] to examine if the SEC is enriched with lethality. There is no enrichment of lethal genes among SEC (*p* = 0.24, OR = 1.08, 95% CI [0.88, 1.30]) and TEC (*p* = 0.83, OR = 0.93, 95% CI [0.79, 1.09]). Finally, using GTEx data, we compared the number of expression quantitative trait loci (eQTLs) associated with SEC and TEC and observed no significant difference in the number of *cis*-eQTLs associated with SEC and TEC (ES = 0, *p* > 0.56; Wilcoxon Rank Sum Test) (Additional file 1: Figure S12). Overall these results highlight that tissue- and cell-specific relevant traits are associated with both SEs and TEs associated genes.

### Enhancer associated genes are connected in a dense interactome

**Fig. 5:**
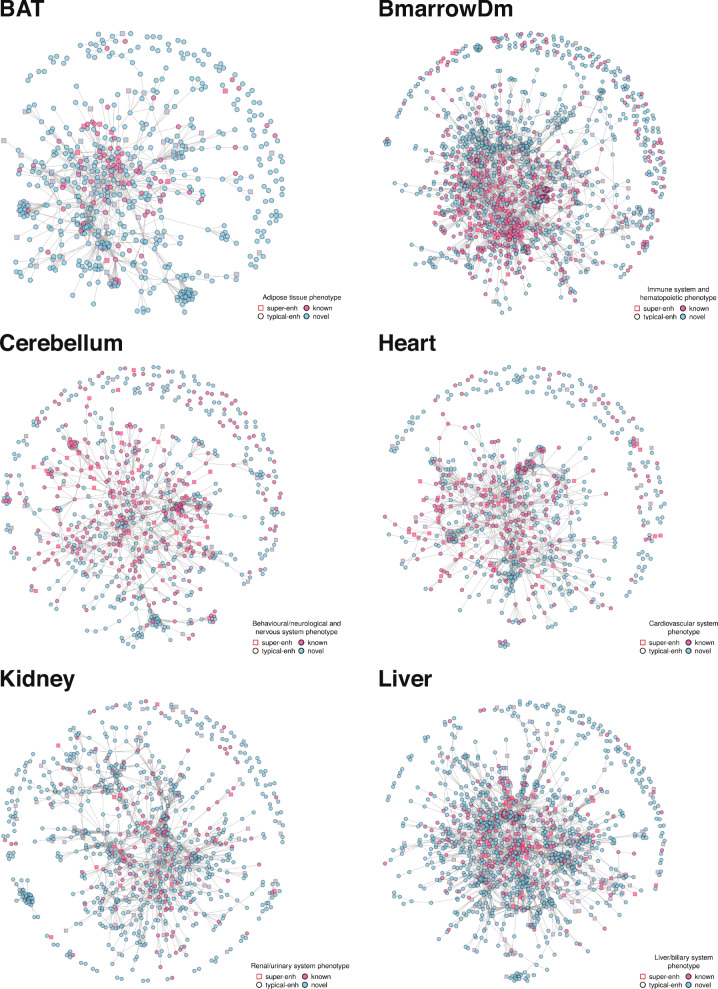
Enhancer associated genes are connected in a dense interactome. The networks display protein-protein interaction maps of enhancer associated genes. Nodes in each network represent enhancer associated genes and edges represent potential protein-protein interactions. Genes associated with tissue-type relevant phenotypes are highlighted in pink and the shape of the node displays SE and TE associated genes (squares: SEC, circles: TEC). See also Additional file 1: Figure S13 and S14

Having shown that enhancer associated genes are enriched for tissue-specific traits, we hypothesised that the proportion of these with no prior phenotypic annotations related to the tissue maybe involved in disease-causing pathways. To identify novel disease-associated genes, we first analysed the protein-protein interactions (PPI) among enhancer-associated genes in each of the 22 tissues, using the STRING database [58]. Then in each network, we identified the genes currently known to be associated with the corresponding tissue-type phenotypic annotations from MGD [59], while the genes with no-prior phenotypic information were labelled as ‘novel’. For each tissue, both the known and unknown disease genes (referred to as known and novel respectively) in the PPI network of enhancer-associated genes are observed to be connected in a remarkably dense interactome (Fig. 5, Additional file 1: Figure S13). Interestingly, the novel genes (blue nodes) are highly connected with the phenotype-associated genes (pink nodes), suggesting a potential functional relationship between them. Simulating these PPI networks with random protein-coding genes showed that novel genes connect significantly more with known phenotype-associated genes, compared to randomly added genes (*p* ≤ 0.016, except thymus *p* = 0.056) (Additional file 1: Figure S14). This outcome demonstrates enhancer associated genes to be potentially engaged in the same functional pathway as the known phenotype genes and therefore, could also be linked with the corresponding phenotypes and ultimately disease causation.

### Preferential transcription factor binding in super-enhancers

Enhancer regions contain many binding sites for TFs which contribute to important tissue-specific functions by regulating the target genes [60]. To investigate transcription factor binding activity within SEs and TEs, with the aim of identifying potential key regulators in each tissue, we used publicly accessible ChIP-Seq data for mouse TFs. For many TFs, the information available on their specific binding in various cell types is rather sporadic, thus we flattened all available ChIP-Seq peaks for each TF into single binding profiles referred to as “cistrome” (see methods). Next, for each cell type, we systematically identified TFs, for which cistrome peaks significantly colocalised with their corresponding active enhancers.

**Fig. 6:**
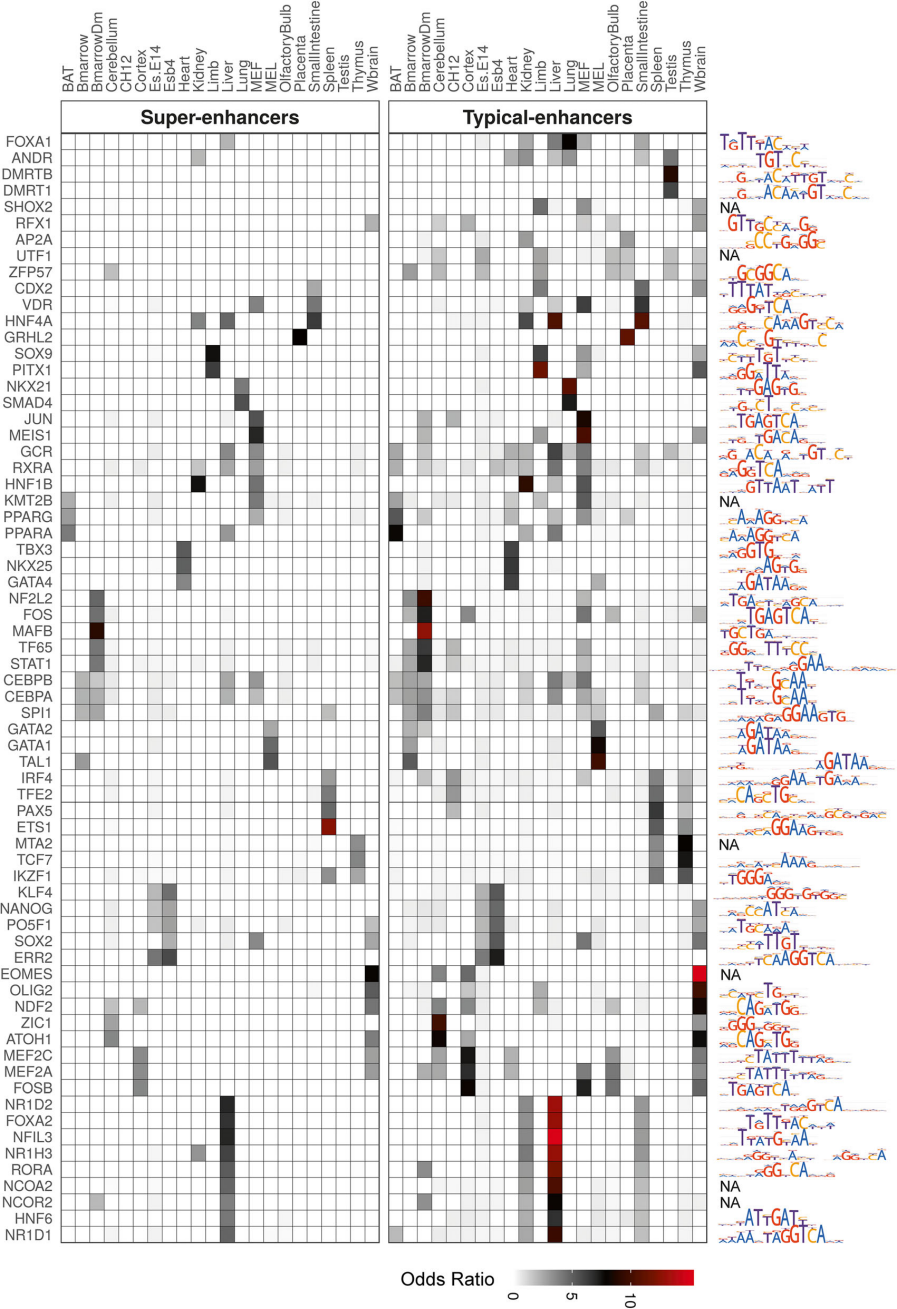
Master regulators enriched in SE and TE constituent enhancers. Heatmap showing the top 3 enriched TFs identified within SEs and TEs in each tissue. The motifs associated with the enriched TFs are shown on the right. NA is shown for TFs with motifs not present in HOCOMOCO v11. The rows of the heatmap are clustered using hierarchal clustering. See also Additional file 1: Figure S15

First, we found that TFs which have significant colocalisation with enhancer elements include regulators known to be implicated in the corresponding tissue-specific regulation (Fig. 6). For example, *Spi1*, with cistrome peaks colocalized with bone marrow enhancers, is implicated in myeloid and B-lymphoid cell development [61]; *Gata4*, with cistrome peaks colocalized with heart enhancers, is involved in myocardial differentiation and function [62]; and *Dmrt1*, with cistrome peaks colocalized with testis enhancers plays a key role in male sex determination and differentiation [63]. Overall, we observed cistrome peaks of 214 TFs (509 TF-tissue pairs) to significantly colocalise with TEs (with OR > 1; corrected *p*-value < 10^− 3^) and 113 TFs (148 TF-tissue pairs) with SEs across all tissues and cell types (Additional file 8). The 214 TFs colocalised with TEs included all the 113 TFs identified for SEs. Second, we observed that some TFs with cistrome peaks significantly colocalised with enhancers are expressed in a tissue-specific manner in the corresponding tissues (Additional file 1: Figure S15a). In total, we identified 56 such TFs with highly tissue-specific expression (*τ*_*exp* − *frac*_ > 0.85) and significant colocalisation with corresponding TEs, and 29 TFs with SEs across all tissues and cell types. Examples of such TFs include *Hnf6* in liver (*τ*_*exp* − *frac*_ = 1), *Nkx2–5* in heart (*τ*_*exp* − *frac*_ = 1), *Gata1* in MEL cells (*τ*_*exp* − *frac*_ = 0.93) and *Neurog2* in brain (*τ*_*exp* − *frac*_ = 0.98).

Overall, TF cistrome peaks were identified to significantly colocalise with both SEs and TEs, but a greater number of TFs were identified to colocalise with TEs compared to SEs. This could be explained by the relatively large number of TEs in the genome. To investigate this further, for each TF with significant enhancer localization, we computed their transcription factor binding site (TFBS) density in SEs and TEs. The TFBS density could be defined as a measure of TFBS clustering in SEs or TEs (see methods). To summarise our analysis, we counted the number of TF-tissue pairs which have significantly greater TFBS density in SEs compared to TEs, and vice-versa for TEs. Overall, we find that SEs have more TF-tissue pairs with higher TFBS density compared to TEs (Additional file 1: Figure S15b). Altogether, this data indicates that although TEs are more often colocalised by TF cistrome peaks, frequency and degree of TFBS clusters is higher in SEs.

### Combinatorial learning approach for phenotype prediction

**Fig. 7:**
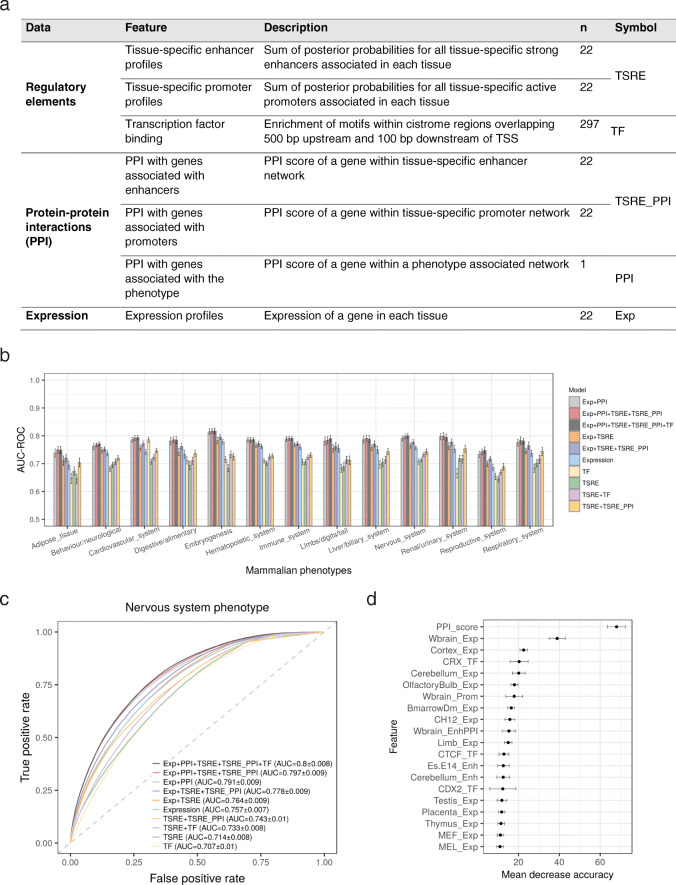
Predicting gene-phenotype associations in mouse. **a** Summary of the various gene features (grouped according to their data sources) used to train the random forest classifier to predict gene-phenotype associations. **b** Bar plot comparing the predictive power of different random forest classifiers across various phenotypes. Error bars denote standard deviation. The classifier trained on all gene features performs the best for majority of the phenotype domains. **c** Receiver operating characteristic (ROC) curves comparing the performance of 10 random forest classifier models applied to predict genes associated with nervous system phenotype. **d** Feature importance chart of the best performing model (Exp + PPI + TSRE+TSRE_PPI + TF) showing the top 20 predictor variables important in nervous system phenotype predictions, as measured by the mean decrease in accuracy (x-axis). The PPI feature was identified to be the most important in predicting genes associated with nervous system phenotype, followed by expression in whole brain and cortex. Exp: expression; Enh: enhancer; Prom: promoter; TF: transcription factor. See also Additional file 1: Figure S16 and S17

Our findings show mouse enhancer associated genes are correlated to a great extent with tissue-specific gene expression as well as phenotypes. We explored the utilisation of this dataset to infer mammalian gene-phenotype associations as has previously been done for protein-protein interaction (PPI) and gene expression data [64–66]. We implemented the random forest classifier to predict gene-phenotype associations from 13 different phenotypic domains, where each domain is relevant to at least one tissue type in our dataset. For this learning approach, we extracted gene features from TSRE profiles, expression data, transcription factor binding sites and protein-protein interaction data in 22 mouse tissues (Fig. 7a) (see methods). For the purpose of training this random forest classifier and maximising its learning process, we combined the SE and TE dataset together and used their constituent enhancers (or tissue-specific enhancers) to calculate the enhancer-associated gene feature. We first trained a random forest classifier on a subset of protein-coding genes using a combination of various gene features as predictor variables and the top level mammalian phenotype terms from MGD as the response variable (true positives), while genes not associated with a phenotype in MGD were considered as true negatives. This model was used to predict gene-phenotype associations in the remaining set of genes not used in the training of the model.

By integrating various features together, 10 combinations were formed, constructing 10 distinct classifiers for each phenotypic domain. The predictive power of each classifier was assessed by generating Receiver Operating Characteristic (ROC) and precision-recall (PR) curves based on 5-fold cross validation, repeated 10 times with different seeds. The classifier trained on all the gene features combined achieved the best performance with a mean AUC-ROC of 0.78 and AUC-PR of 0.27 across all the phenotype domains (Fig. 7b, Additional file 1: Figure S16, Additional file 9). However, high precision recall rates (AUC-PR > 0.35) are observed in phenotypes with a high number of known mammalian phenotype annotation counts in MGD (such as behavioural/neurological, nervous system, cardiovascular, immune and hematopoietic system, see Additional file 1: Figure S17). Focusing on predicting gene-phenotype associations within the nervous system domain, the classifier trained on all the gene features achieved the greatest mean AUC-ROC of 0.80 and AUC-PR of 0.42 (Fig. 7c). The PPI score with genes known to be associated with nervous system phenotype was identified to contribute the most in predicting nervous system gene-phenotype associations, followed by expression data in whole brain and cortex (Fig. 7d). In fact, PPI data is the most informative and the main contributor to the performance of these classifiers in all the 13 phenotypes. While the models trained solely on regulatory features have limited predictive power, they improved the performance of models when integrated with other features, suggesting regulatory data are a useful addition for modelling mammalian phenotypes.

In order to evaluate the validity of the predictions from the model, we investigated the novel gene-phenotype predictions made by these classifiers. The predictions classified as incorrect are based on the current knowledge of gene-phenotype associations, but it is possible that there are no, or little, prior knowledge about particular gene function, and thus are novel. This also leads to undermining the true predictive power of a classification model. For such reasons, the top false-positive predictions are most interesting as they could provide new hypotheses about gene function. To systematically examine the top false-positive predictions (prediction score ≥ 0.90) in each phenotype domain, we used the Open Targets Platform [67] and the DisGeNET discovery platform [68] which links potential novel genes to diseases via evidence based on genetic associations, somatic mutations, animal models, expression, pathways, drugs and text mining from literature. We identified that ~ 75% (495/659) of the false-positive predictions examined (see methods) with Open Targets and ~ 63% (338/539) with DisGeNET could be potentially associated with the corresponding disease (Additional file 1: Figure S18) and hence, could serve as potential novel disease targets. For example, out of the 76 top scoring false-positives (prediction score ≥ 0.90) examined for nervous system phenotype, 72 could be associated with nervous system disease (*p* = 5.00 × 10^− 9^) based on evidence integrated from a range of data sources by Open Targets platform. Additional file 10 provides these novel predictions for each phenotype and the evidence supporting their association with the corresponding diseases.

## Discussion

Regulatory elements have been identified as active in a plethora of cell types and tissues, however there is limited understanding about their relationship to overall gene function and the resulting gene-phenotype relationships. To gain insights into the mammalian regulatory landscape and its potential impact on phenotypic outcome, we focused our analysis on tissue-specific enhancers. By generating a catalogue of super, typical and weak enhancers in multiple mouse tissues we systematically investigated their roles in gene function. From multiple aspects such as gene expression, PPI networks and phenotypes, our study now provides evidence that SE and TE associated genes share common phenotypic outcomes even though their expression profiles and overall numbers in the genome differ.

SEs are comprised of dense enhancer clusters spanning large genomic regions and are associated with master transcription factors and other key cell identity genes [21, 31]. We observed that compared to TEs, SEs consists of a large number of constituent enhancers, however, the mechanistic mode of action of these individual constituent elements is not well understood. It remains unclear whether the constituent enhancers exert an additive or a more complex cooperative effect on target gene expression. Using our genome-wide enhancer maps, we sought to examine the effect of constituent enhancer density on the total-expression of genes at a genome-wide scale. Our results show that globally, total-expression levels of genes are weakly correlated with the number of constituent enhancers. The constituent enhancer density explains only a small fraction of the variation in gene expression, indicating a complex rather than a linear additive relationship between constituent enhancers and target gene expression. Not all constituent enhancers appear to contribute to the transcriptional output with the same strength, suggesting some constituent enhancers may make small contributions therefore helping to fine tune the expression patterns of their associated genes. This observation is consistent with previous in vivo experiments showing the effect of deleting individual SE constituents on target gene expression is highly variable [50, 69, 70]. SE constituents have more chromatin interactions among themselves [47], suggesting these constituent enhancers may have an effect on one another’s contribution towards the target gene transcriptional activity. However, we cannot rule out the possibility that some constituent enhancers may have a redundant function in transcriptional activation [71]. It should be noted this study is a computational prediction and has limitations. In order to accurately calculate the impact of constituent enhancers on target gene expression, it is important to know which constituent enhancers are real and/or active alongside the gene(s) they regulate.

Prior research has thoroughly investigated the role of SEs in complex traits, showing that disease-causing SNPs are more enriched in SEs of disease-relevant cell types [21, 40, 41]. However, little research has been conducted to systematically examine the effect of SEs and TEs on diseases. Here, we investigated the mammalian phenotype and disease associations of SE and TE associated genes. We identified that both the SEC and TEC are significantly enriched in phenotypes and diseases in the corresponding tissue-types (Fig. 3), emphasising that phenotypes are governed by tissue-specific enhancers. Using phenotyping data from knockout mouse lines of enhancer associated genes, we show that there is no significant difference in severity and breadth of phenotypes produced from knockouts of SEC and TEC (Fig. 4, Additional file 1: Figure S11), which underscores the importance of both enhancer classes in disease causation. In addition, no difference in enrichment of mouse essential genes and number of eQTL associations was identified among SEC and TEC. Overall, we did not find any significant contrast between the potential phenotypic impact of SEC and TEC, suggesting that functional testing of all enhancers irrespective of categories is fundamental in making any conclusions about their functional significance and phenotypic impact. Although the majority of key cell identity genes and TFs are associated with SEs, the ‘peripheral’ genes associated with TEs appear to equally contribute towards disease aetiology. A possible explanation to this surprising result is the existence of an ‘omnigenic’ architecture [72] where regulatory networks are densely inter-related such that TE associated genes expressed in disease relevant cell types can collectively impact the regulation of key cell identity genes. To this end, we hypothesised that tissue-specific enhancer associated genes are components of protein complexes involved in aberrant disease-causing biochemical processes and could be potential therapeutic targets. Our PPI analysis show that enhancer associated genes with no prior corresponding tissue-type phenotypic associations preferentially interact with known phenotype-associated genes. This observation suggests that these enhancer associated genes could serve as novel targets for diseases.

Finally, using a machine learning approach, we systematically evaluated the capability of TSREs and other molecular properties to predict gene-phenotype associations in mouse (Fig. 7). By comparing classifiers trained on different gene features, we found the classifier with all the gene features combined performs the best to predict gene-phenotype associations. Our results also reveal that PPI data have a high predictive capacity to infer mammalian gene-phenotype associations, while regulatory data provides a modest but additive source of information. Further examination of the top scoring false-positive predictions shows their promising application in generating hypothesis about gene function and in identification of potential novel disease targets. Such prediction models can assist in prioritising genes in mouse knockout and genome editing studies. They could also help in selecting the most relevant phenotyping procedures (which often involves costly assays) for transgenic mice models.

## Conclusion

In this study, we systematically characterised different enhancer types with the goal of investigating their roles in gene function. We found that super- and typical-enhancers have different effect on gene expression, but both are preferentially associated with relevant tissue-type mammalian phenotypes and human diseases. We show that genes associated with super- and typical-enhancers exhibit no difference in phenotype effect size or pleiotropy suggesting they share common phenotypic outcomes. Our findings in a diverse range of mouse tissues present opportunities for molecular experiments to investigate regulatory mechanisms in mouse models of human diseases.

## Methods

### Learning chromatin states and segmentation of the mouse genome

First, the ChIP-Seq data for histone H3 lysine 4 monomethylation (H3K4me1), histone H3 lysine 4 trimethylation (H3K4me3) and histone H3 lysine 27 monoacetylation (H3K27ac) in 22 mouse tissues and cell lines were collected from ENCODE project (LICR lab) in the form of sequence alignments (BAM files mapped to mm9 mouse genome). The 22 epigenomes include 14 adult tissues: BAT (brown adipose tissue), bone marrow, cerebellum, cortex, heart, kidney, liver, lung, olfactory bulb, placenta, small intestine, spleen, testis and thymus; 2 embryonic tissues: limb and whole brain; and 6 cell lines: bone marrow derived macrophage, CH12 (B-cell lymphoma, GM12878 analog), Esb4 (mouse embryonic stem cells), Es-E14 (mouse embryonic stem cell line E14), MEF (mouse embryonic fibroblast), MEL (leukemia, K562 analog). Next, we used a multivariate hidden Markov model called ChromHMM to integrate all the ChIP-Seq data and summarise into easily illustratable annotations. The chromatin states were jointly learned across 22 mouse tissues using default parameters. Several HMM models were produced consisting of 4–8 chromatin states and identified the 6 state model to provide sufficient resolution to isolate biologically meaningful chromatin states. The resulting chromatin states were then annotated based on the biological significance of the frequencies of combined histone marks. Using this approach, potential active promoter (404,016), weak promoter (647,185), strong enhancer (1,075,608) and weak enhancer (2,068,844) annotations were mapped across 22 mouse tissues and cell types. To validate our predicted promoter states (states 1 and 2), we compared 217,678 unique non-overlapping promoters to 22,707 known protein coding genes (mm9 ensembl genes v67; 10 kb upstream, 100 bp downstream of TSS) and recovered 81.66% of known promoters. Similarly, to validate the strong enhancer predictions (state 4), we compared 386,222 unique non-overlapping enhancers to 363 experimentally validated VISTA mouse enhancers and recovered 91.18% of VISTA enhancers from our predictions. Chromatin states with < 0.95 posterior probability were filtered resulting in 923,791 strong enhancer (state 4); 309,581 active promoter (state 2); 2,531,993 weak enhancer (state 6); and 427,251 weak promoter (state 1) high confidence annotations respectively.

### Identifying tissue-specific regulatory elements

To identify tissue-specific regulatory regions across the 22 tissues, we implemented the Tau method which has been previously used to detect tissue-specific expression [43, 44]. Tau is a measure of tissue specificity index which takes into account number of tissues and normalised expression in each tissue and outputs a score for each gene. To implement this method, we constructed matrices of chromatin state posterior probabilities for strong enhancers, active promoters, weak enhancers and weak promoters with dimension *n × s*, where *n* is the number of regulatory elements and *s* is the number of tissues (i.e. 22). Each row of the matrix is a genomic location of the regulatory element (200 bp in length) and columns represents its posterior probability across all the tissues. The matrices were filtered such that only the regulatory elements with a posterior probability ≥0.95 in at least one tissue were retained. The Tau score for each regulatory element was calculated by the following equation:

1 $$ {\tau}_{reg}=\frac{\sum \limits_{i=1}^N\left(1-\hat{x_i}\right)}{N-1};\hat{x_i}=\frac{x_i}{\max \left({x}_i\right)} $$

where *N* is the number of tissues and *x*_*i*_ is the posterior probability value. Using the thresholds suggested in [43], the regulatory elements were categorised into low (*τ*_*reg*_ ≤ 0.15), intermediate (0.15 < *τ*_*reg*_ < 0.85), high (0.85 ≤ *τ*_*reg*_ < 1) and absolute tissue-specific (*τ*_*reg*_ = 1).

### Correlating TSREs with DNaseI hypersensitive sites

For DNasel accessible regions, we collected DNasel hypersensitivity sites (DHS) in 11 tissues (Cerebellum, CH12, Es-E14, Heart, Kidney, Liver, Lung, MEL, Spleen, Thymus, Wbrain) from ENCODE (UW lab) in the form of hotspots. The mean of DNaseI signal was computed wherever multiple replicates were available within ENCODE. The genomic coordinates of tissue-specific enhancer and promoter elements were compared with DNaseI hypersensitive hotspots using BEDTools [73] and the DNaseI signal in each tissue or cell line was extracted. We restricted the extraction of DNaseI signal to cases where 100% of the enhancer or promoter region overlapped the DHS hotspot, otherwise no DNaseI activity was assumed and a value of “0” was assigned to that enhancer or promoter. This resulted in a matrix of DNaseI signal corresponding to the posterior probability matrix of tissue-specific enhancers and promoters. To quantify the concordance between TSREs (tissue-specific enhancers and promoters) and DHS, Pearson’s correlation between posterior probability of their respective chromatin state and the corresponding DNasel signal was calculated. The pairwise correlations between the tissues were visualised in a heatmap and rows and columns were ordered based on hierarchical clustering (Additional file 1: Figure S2b and S2c).

### Distinguishing super-enhancers from typical-enhancers

To identify SEs in mouse, we implemented an approach similar to previously used by [31]. Using the ROSE algorithm, tissue-specific enhancers within a distance of 12.5 kb were stitched together into cohesive units and ranked based on their H3K27ac signal. A TSS exclusion size of 2000 bp was used to exclude tissue-specific enhancers within ±2 kb of a known TSS to remove any promoter bias. The algorithm calculates a threshold of the inflection point for H3K27ac signal. The stitched cohesive units with H3K27ac signal higher than the estimated threshold are defined as SEs while the remaining cohesive units are termed as TEs.

The metagene profiles of mean H3K27ac signal across all the SEs and TEs (Fig. 1d, Additional file 1: Figure S3) were generated using ngs.plot [74]. Metagene plots are centered on the enhancers and display average ChIP-Seq read density over all the enhancer regions and surrounding windows of 2 kb. For visual comparison between profiles of SEs and TEs in a tissue, the range of the y-axis were synchronised. For comparing the H3K4me1, H3K27ac and DNAseI hypersensitivity signal over the stitched enhancers (Additional file 1: Figure S4), the read density over these regions was calculated in reads per million (rpm). For H3K4me1 and H3K27ac ChIP-Seq signal, the input control density was subtracted in rpm. The read density for each feature was then normalized by dividing the signal at each enhancer by the maximum signal in each feature. The stitched enhancers for each feature on x-axis are ranked according to the H3K27ac ChIP-Seq signal.

### Effect size calculation

The non-parametric effect size (ES) was calculated as the difference in medians of the two groups divided by the pooled median absolute deviation (MAD). The following formula was used:

$$ ES=\frac{Median_1-{Median}_2}{MAD_{pooled}};{MAD}_{pooled}=\sqrt{\frac{MAD_1^2+{MAD}_2^2}{2}} $$

### Associating TSREs to potential target genes

We used GREAT [48] to associate tissue-specific regulatory elements to potential target genes in each tissue. In cases where GREAT predicted multiple target genes for a particular TSRE, the nearest gene was selected as the primary predicted target for all further downstream analysis. GREAT was run using default parameters on mm9 assembly and the whole genome was selected for control background regions. The coordinates of TSREs and their associated genes in all tissues are provided in Additional file 2. To examine the consistency of our enhancer-gene assignments with other datasets, we compared them to previously reported topologically associated domains (TADs) [49] and enhancer-promoter units (EPUs) [6] in mouse. The enhancer-gene pairs across the 22 tissues were merged together for this comparison. The TADs (in mESC and cortex) were compared to the enhancer-gene pairs to examine if the enhancer-gene pair overlaps the same TAD (Additional file 3). Only the cases where both enhancer and its associated gene overlapped a TAD were used. We identified 96.62 and 93.57% of our enhancer-gene pairs to be in the same TADs annotated in cortex and mESC respectively. A similar comparison was done with EPUs which revealed 87.23% of our predicted enhancer-gene pairs to be in the same EPU.

### Expression analysis of enhancer associated genes

For investigating the expression of enhancer associated genes, RNA-Seq data for all 22 tissues and cell lines was collected from ENCODE as read alignments (BAM files). Data for cell lines CH12 and Es-E14 was collected from Standford/Yale lab while rest of the data was retrieved from LICR lab. From the BAM files, the read counts over all genes (mm9, ensembl v67) were quantified using HTSeq [75] and expression of each gene was calculated in RPKM (Reads Per Kilobase of transcript per Million mapped reads) in each tissue/cell line. A mean RPKM value was calculated for multiple biological replicates from ENCODE.

To examine the relationship between enhancers and expression of their target genes, data from all 22 tissues was combined into gene-tissue pairs and grouped into three classes based on their enhancer association: (1) gene-tissue pairs associated with SEs (SEC); (2) gene-tissue pairs associated with TEs (TEC); and (3) gene-tissue pairs associated with weak/poised enhancers (WEC). In order to quantify tissue-specific expression of target genes, we calculated the tissue specificity index for each gene using the Tau method described earlier. We constructed a matrix of expression values with dimensions *t* × *s*, where *t* is the total number of genes and *s* is the number of tissues/cell lines. Genes not expressed in any tissue were deleted from the matrix leaving genes expressed in at least 1 tissue. The RPKM values were log2 transformed and quantile normalised (QN) (using the normalize.quantiles function in preprocessCore R package) to allow easier comparison of gene expression across tissues. Genes were then sorted by ascending QN value and divided into deciles of equal density and placed into 10 bins. The lowest decile (lowest 10% of genes by QN value) was placed in bin 1, the next lowest was placed in bin 2, and so on until the top 10% of QN values were placed in bin 10. The Tau value (*τ*_*exp*_) for each gene was calculated as:

2 $$ {\tau}_{exp}=\frac{\sum \limits_{i=1}^N\left(1-\hat{y_i}\right)}{N-1};\hat{y_i}=\frac{y_i}{\max \left({y}_i\right)} $$

where *N* is the total number of tissues, *y*_*i*_ is the normalised expression bin profile component of the gene in tissue *i*. In order to associate *τ*_*exp*_ values to tissues, Tau-fraction (*τ*_*exp* − *frac*_) for each gene in every tissue was calculated as $\frac{\tau_{exp}\times {r}_i}{M}$ where *r*_*i*_ is the expression of the gene (in RPKM) in tissue *i* and *M* is the maximum expression of the gene (in RPKM) across all the tissues. Based on *τ*_*exp* − *frac*_ score, the genes were categorised into low (*τ*_*exp* − *frac*_ ≤ 0.20), intermediate (0.20 < *τ*_*exp* − *frac*_ < 0.85) or high (*τ*_*reg*_ ≥ 0.85) tissue-specific expression in the corresponding tissues. Housekeeping genes were identified based on a strict *τ*_*exp*_ threshold. Genes with low *τ*_*exp*_ score (≤0.20) are uniformly expressed across all the tissues and were considered to be housekeeping genes. We identified 1252 housekeeping genes using this threshold out of which 1171 were protein-coding genes.

To visualise the distinct number of enhancer tissue-types calculated for each enhancer-associated gene (Fig. 2f), we generated two binary matrices for SEC and TEC in 22 tissues. The rows in the matrix represented enhancer associated genes and columns represented different tissues. A value of “1” or “0” was assigned to the cells in the matrix depending on if the gene was identified to be associated with the enhancer of that tissue or not respectively. The heatmaps in Fig. 2f were first sorted on the number of enhancer tissue-types and then sorted by the order of tissues across the columns.

### GO, mammalian phenotype and disease enrichment analysis

To investigate the molecular functions and biological processes linked with enhancer associated genes, we combined the SE and TE associated genes across the 22 tissues to make two unique lists. This resulted in 3617 genes to be only associated with SEs and 11,437 genes to be only associated with TEs. These gene sets were then used for GO enrichment analysis using ToppGene suite [76] (Additional file 7). The enrichment of mammalian phenotypes and human diseases was calculated individually in each tissue using the ToppFun tool in ToppGene suite. The enrichment of housekeeping genes among SEC and TEC was calculated using Fisher’s exact test. For background, total number of protein coding genes in our annotation set was used. SEC is significantly depleted for housekeeping genes (155/3617; *p* = 0.012, OR = 0.82) while TEC is enriched (686/11,437; *p* = 2.7 × 10^− 11^, OR = 1.49).

### Mouse gene knockout data

The mouse phenotyping data of enhancer associated gene knockouts was extracted from IMPC (International Mouse Phenotyping Consortium). All the statistically significant genotype-phenotype associations and their phenotyping data in IMPC release version 5.0 were used. This version compromised of phenotype data for 3323 gene knockouts, with 2900 genes significantly associated with at least one phenotype attribute (*p* < 10^− 4^). To quantify the severity of phenotypes, we used the percentage change value from each procedure. The percentage change is normalised effect size, which is scaled to make it comparable across various procedures and parameters [77]. The percentage change between SE and TE associated genes was compared for several standardised phenotyping procedures. The phenotype procedure protocols are described in IMPReSS (https://www.mousephenotype.org/impress). All the parameters within a procedure were grouped together for this analysis. For computing the enrichment of mouse essential genes in SEC and TEC, genes producing a lethal homozygous knockout (960 genes out of 2900) were used.

### GTEx expression quantitative trait loci

The official set of GTEx v8 significant variant-gene associations based on permutations and conditionally independent eQTLs mapped using stepwise regression were used for the analysis. For each gene in SEC and TEC, we extracted and counted the total number of eQTL associations. This analysis was performed in the following tissues: cerebellum, cortex, heart, liver, lung, small intestine, spleen and testis.

### Known gene-phenotype associations

All the gene-phenotype associations in mouse were extracted from MGD. The Mouse Phenotypic Alleles report (MGI_PhenotypicAllele.rpt) was collected from MGD on 14th June 2017.

### Protein-protein interaction maps

The predicted protein-protein interactions among the genes of interest were extracted from the STRING database [58] using the R package STRINGdb. A score threshold of 900 was implemented to extract potential interactions with the highest confidence and reduce false-positives. These interaction maps were visualised as networks using the iGraph package in R. The known gene-phenotype associations (from MGD) in the network were labelled as “known” while the remaining genes were marked as “novel”. A permutation test was performed to identify if the observed number of interactions between known and novel genes are more than what we would expect by random (Additional file 1: Figure S14). We added randomly selected protein-coding genes equal to the number of genes known to be associated with phenotypes in the network and extracted their interactions from STRING. The number of interactions (edges) between randomly added genes and known phenotype genes were then counted. This was repeated 1000 times to produce a distribution of expected number of edges and the *p*-value was calculated as $$ p=\raisebox{1ex}{$y$}\!\left/ \!\raisebox{-1ex}{$N$}\right. $$, where *y* is number of permuted random-known edges greater than the observed novel-known edges and *N* is the total number of items in our distribution (i.e. 1001).

### Cistrome data

For the analysis of transcription factor binding sites colocalised with different enhancer sets, we used a cell type independent cistrome, the general genomic map of regions bound by particular TFs in any cell type [78]. The cistrome is based on uniformly reprocessed ChIP-Seq data from the GTRD database [79] across all the cell types and conditions. The cistrome regions are classified into four reproducibility categories (A,B,C,D): A - regions supported by ChIP-Seq data from two different experimental data sets (at least one was accompanied by control data) and different ChIP-Seq peak calling tools; B - regions supported by peak calls from two different experimental data sets (at least one was accompanied by control data); C - regions supported by peak calls from a single experimental data set with control data and different peak calling tools; D - all other reproducible regions (supported by more than one peak). A and B categories were taken into the analysis by default. For TFs with a limited number of ChIP-Seq data sets, we added regions from C and D categories when it was necessary to get at least 100 peaks. As an additional filter for cistrome, we used TF binding motifs from HOCOMOCO to annotate motif occurrences in cistrome regions with SPRY-SARUS [80] using the default motif *p*-value threshold of 5 × 10^−4^ [81] and then discarded cistrome segments without motif occurrences.

### Enrichment of TFBS in SEs and TEs

To calculate the enrichment of TF binding within SE and TE constituents, we first merged the neighbouring constituent enhancers within 400 bp into prolonged extended enhancer segments in each tissue. These extended enhancer segments were then used to generate the control regions; more precisely, for each enhancer segment of length L, we located two segments (enhancer shades) of length L, one at 100 L upstream and the other at 100 L downstream. This produced a set of control segments of the same lengths and similar global genomic context as the enhancer segment under study. We checked if any control segments overlapped other constituent enhancers, but such cases contributed only 1–2% of the total number of control regions and were safely ignored. The extended enhancer segments and control regions were then intersected with the cistrome peaks of each TF and split into two groups; overlapping (if least 1 bp overlapped) and non-overlapping with the cistrome. The Fisher’s exact test on 2 × 2 contingency tables was used to assess the statistical significance of TF cistrome peaks overlapping constituent enhancers (SE or TE) versus control regions (Additional file 8). The resulting *p*-values were corrected for multiple testing using Bonferroni correction. Note that the cistrome segments of a TF could significantly colocalise with enhancers in several different cell types, therefore, we counted the number of significant enrichments as TF-tissue pairs. We also performed the analysis with only the cistrome segments that contain high scoring motif hits from HOCOMOCO. The results were very similar to the analysis where all cistrome segments were considered; about 10% of TFs did not have known binding motifs, and for TFs with known motifs, about 90% of significant TF-tissue pairs were independent from whether the motifs were considered or not (Additional file 8).

### TFBS density analysis

To calculate the TFBS density of each TF, we intersected each enhancer element with the TF cistrome peaks. Within these overlapping regions, we predicted the binding motif occurrences of the corresponding TF using HOCOMOCO-v11 motifs. In cases where HOCOMOCO contained multiple motif models for a single TF, all motifs were used and the binding sites exceeding the cistrome *p*-value threshold of 0.0005 were retained. Density was calculated as the total genomic coverage of motifs (in bp) divided by the total coverage of enhancer-cistrome intersection (in bp). We calculated densities for only those enhancers (constituent enhancers of SEs or TEs) which had at least one motif occurrence in its intersection with the cistrome. The Wilcoxon Rank Sum Test was then used to compare the TFBS densities of TF-tissue pairs in SEs and TEs (each TF-tissue pair was compared individually between SEs and TEs). The non-corrected *p*-values were used to order the TF-tissue pairs by their level of TFBS density disparity between SEs and TEs. The TF-tissue pairs were grouped into bins based on their *p*-value and the number of TF-tissue cases where its TFBS density was more in SEs compared to TEs, or vice versa, were counted (Additional file 1: Figure S15b).

### Predicting gene-phenotype associations

To predict mammalian gene-phenotype associations, features were extracted from TSREs, expression, transcription factor binding and PPI data for all protein-coding genes. From the TSRE profiles across 22 tissues, strong-enhancers and active promoters associated with each protein-coding gene were extracted. A score representing the tissue-specific enhancers and promoters in each tissue was computed as ${S}_{gt}=\sum \limits_{i=1}^N(P)$, where *S*_*gt*_ is the score of gene *g* in tissue *t*; *N* is the total number of strong enhancer or active promoter elements associated with gene *g* in tissue *t*; and *P*_*i*_ is the posterior probability of the associated strong enhancer or active promoter element emitted by the ChromHMM model. The RPKM values for each gene, quantified using ENCODE’s RNA-Seq data in 22 tissues were used as a feature for expression data. For TF binding associated with each gene, we first we selected all cistrome regions overlapping − 500 bp and + 100 bp of TSS (for each gene, we considered all transcripts from gencode vM15). Then, we calculated the -log10(*p*-value) of HOCOMOCO motif hits within these cistrome regions (aggregating over all motifs if there were multiple models for a particular TFBS). The respective values for each TF were taken as the TFBS features. The final set of the TFBS features covered all TFs for which we had the ChIP-Seq cistrome peaks and a binding motif model (*n* = 297). For PPIs, all the protein interactions in mouse were collected from STRING database version 10.5. For a gene *g*, its PPI connectivity with all strong enhancer and active promoter associated genes in tissue *t* was calculated as ${PPI}_{gt}=\sum \limits_{i=1}^N(I)$, where *N* is the total number of enhancer or promoter associated genes in tissue *t* and *I*_*i*_ is the combined interaction score between gene *g* and *i*^*th*^ gene. Similarly for each gene, its PPI connectivity with all genes known to be associated with the phenotype domain to be predicted was computed as ${PPI}_{g- phen}=\sum \limits_{i=1}^M(I)$, where *I* is the interaction score and *M* is the total number of known phenotype associated genes from MGD.

The random forest classifier was implemented in R using randomForest and caret package [82]. We sought to predict gene-phenotype associations from 13 different phenotypes relevant to at least one tissue type in our dataset. The known gene-phenotype associations from MGD (top level MP annotations) served as true-positives for the classifier models. The random forest classifier was trained on a subset of genes (using default parameters), where features described above were used as predictor variables and phenotype calls from MGD as the response variable. This model was used to predict gene-phenotype associations in the remaining set of genes not used in the training of the model. The preProcess function in caret was used to centre and scale all the gene features. Down-sampling was employed on the training data to avoid the impact of class imbalance on model fitting. Model optimisation across these parameters was performed using k-fold cross validation technique, to choose the model with the best ROC (parameters used: method = “repeatedcv”, number = 5, repeats = 5, metric = “ROC”). In order to compare the predictive capability of various gene features, 10 different models with different gene feature combinations were built for each phenotype domain (130 models in total). Each of these classifier was assessed by generating ROC and PR curves based on 5-fold cross validation repeated 10 times. The cross validation results were then averaged for comparison and reporting purposes. The top false positives hits (prediction probability ≥0.90) were examined using the Open Target Platform and the DisGeNET discovery platform to validate the novel predictions. Predictions from only those phenotype domains were investigated which had a corresponding disease class in Open Targets and DisGeNET platform. As a result, predictions from 12 phenotypes were examined with Open Targets platform and predictions from 9 phenotypes were examined with DisGeNET (see Additional file 10).

## Supplementary information

**Additional file 1: Figure S1.** Chromatin state segmentation and characterisation across 22 mouse tissues. **Figure S2.** Overview of tissue-specific regulatory elements in the mouse genome. **Figure S3.** H3K27ac activity within SEs and TEs. **Figure S4.** Enrichment of chromatin marks over stitched cohesive enhancer units. **Figure S5.** Chromatin activity within SE and TE constituent enhancers. **Figure S6.** Region-gene associations of regulatory elements. **Figure S7.** Relationship between enhancer activity and their target gene expression. **Figure S8.** Impact of constituent enhancer density on target gene expression. **Figure S9.** Enhancer usage switch associated with genes within SEC and TEC with multiple enhancer tissue-types. **Figure S10.** Genomic view of genes demonstrating enhancer usage switch. **Figure S11.** Breadth of phenotypes associated with SE and TE gene knockouts in mouse. **Figure S12.** Number of eQTLs associated with genes within SEC and TEC. **Figure S13.** Protein-protein interaction maps of enhancer associated genes. **Figure S14.** Protein-protein interaction simulations. **Figure S15.** Transcription factor binding within SE and TE constituents. **Figure S16.** Performance of random forest classifiers to predict mammalian gene-phenotype associations. **Figure S17.** Precision and recall of classifiers used to predict gene-phenotype associations. **Figure S18.** Evaluation of top-scoring false-positives using the Open Targets platform. **Table S1.** Mammalian phenotype and human disease ontology terms enriched in genes associated with weak-enhancers.

**Additional file 2.** List of genes associated with SEs (SEC) and TEs (TEC) in 22 tissues.

**Additional file 3.** Comparison of enhancer-gene pairs with TADs and EPUs.

**Additional file 4.** Enhancer tissue-type association of SEC and TEC.

**Additional file 5.** Enhancer usage switch scores of genes within SEC and TEC.

**Additional file 6.** Gene Ontology enrichment of genes within SEC and TEC.

**Additional file 7.** Mouse phenotype and human disease enrichment of genes within SEC and TEC in 22 tissues.

**Additional file 8.** Enrichment of TF cistrome peaks within SE and TE regions.

**Additional file 9.** Performance metrics of all random forest classifiers.

**Additional file 10.** Exploration of top scoring predictions and the evidence supporting their association with the corresponding diseases.

## Data Availability

The datasets supporting the conclusions of this article are included within the article and its additional files. Source code of the analysis can be found here: https://github.com/MRC-Harwell/SuperEnhancers

## Abbreviations

TFs: Transcription factors
DHS: DNaseI hypersensitive sites
IMPC: International mouse phenotyping consortium
SEs: Super-enhancers
TEs: Typical-enhancers
TSREs: Tissue-specific regulatory elements
TADs: Topological associated domains
EPUs: Enhancer-promoter units
TSS: Transcription start site
SEC: Super-enhancer class
TEC: Typical-enhancer class
WEC: Weak-enhancer class
ES: Effect size
GO: Gene ontology
OR: Odds ratio
PPI: Protein-protein interactions
TFBS: Transcription factor binding site
ROC: Receiver operating characteristic
PR: Precison-recall
AUC: Area under the curve
