# Supplementary material for: A holistic view of mouse enhancer architectures reveals analogous pleiotropic effects and correlation with human disease
Source: BMC Genomics. 2020 Nov 2;21:754. doi: 10.1186/s12864-020-07109-5 (PMC7607678; doi:10.1186/s12864-020-07109-5)
Supplement: Supplementary file 1 — Additional file 1: Figure S1. Chromatin state segmentation and characterisation across 22 mouse tissues. Figure S2. Overview of tissue-specific regulatory elements in the mouse genome. Figure S3. H3K27ac activity within SEs and TEs. Figure S4. Enrichment of chromatin marks over stitched cohesive enhancer units. Figure S5. Chromatin activity within SE and TE constituent enhancers. Figure S6. Region-gene associations of regulatory elements. Figure S7. Relationship between enhancer activity and their target gene expression. Figure S8. Impact of constituent enhancer density on target gene expression. Figure S9. Enhancer usage switch associated with genes within SEC and TEC with multiple enhancer tissue-types. Figure S10. Genomic view of genes demonstrating enhancer usage switch. Figure S11. Breadth of phenotypes associated with SE and TE gene knockouts in mouse. Figure S12. Number of eQTLs associated with genes within SEC and TEC. Figure S13. Protein-protein interaction maps of enhancer associated genes. Figure S14. Protein-protein interaction simulations. Figure S15. Transcription factor binding within SE and TE constituents. Figure S16. Performance of random forest classifiers to predict mammalian gene-phenotype associations. Figure S17. Precision and recall of classifiers used to predict gene-phenotype associations. Figure S18. Evaluation of top-scoring false-positives using the Open Targets platform. Table S1. Mammalian phenotype and human disease ontology terms enriched in genes associated with weak-enhancers. [file 12864_2020_7109_MOESM1_ESM.pdf]

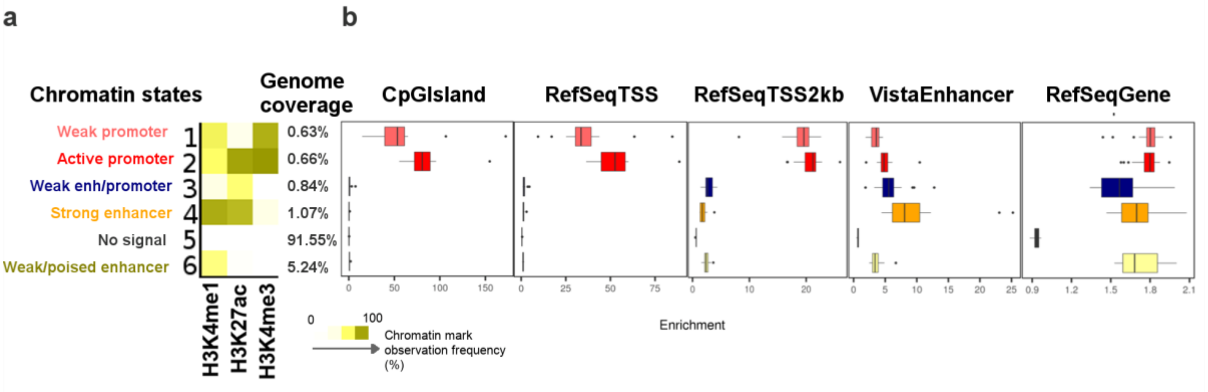

Figure S1: Chromatin state segmentation and characterisation across 22 mouse tissues. (a) Heatmap showing the 6 state model emission parameters learned jointly across tissues by applying ChromHMM. Columns show the various histone marks used in the model, rows show the chromatin states with their potential functional annotation (interpreted from combinations of histone marks in each state), colour denotes the frequency with which a histone mark is observed in the genome, and genome-wide coverage of each state is shown on the right. (b) Box plots showing the enrichment of each chromatin state over functional annotations across all the tissues (in order: CpG islands, TSS, within 2 kb from a TSS, Vista enhancers and genes). A higher enrichment value denotes a higher abundance of the chromatin state.

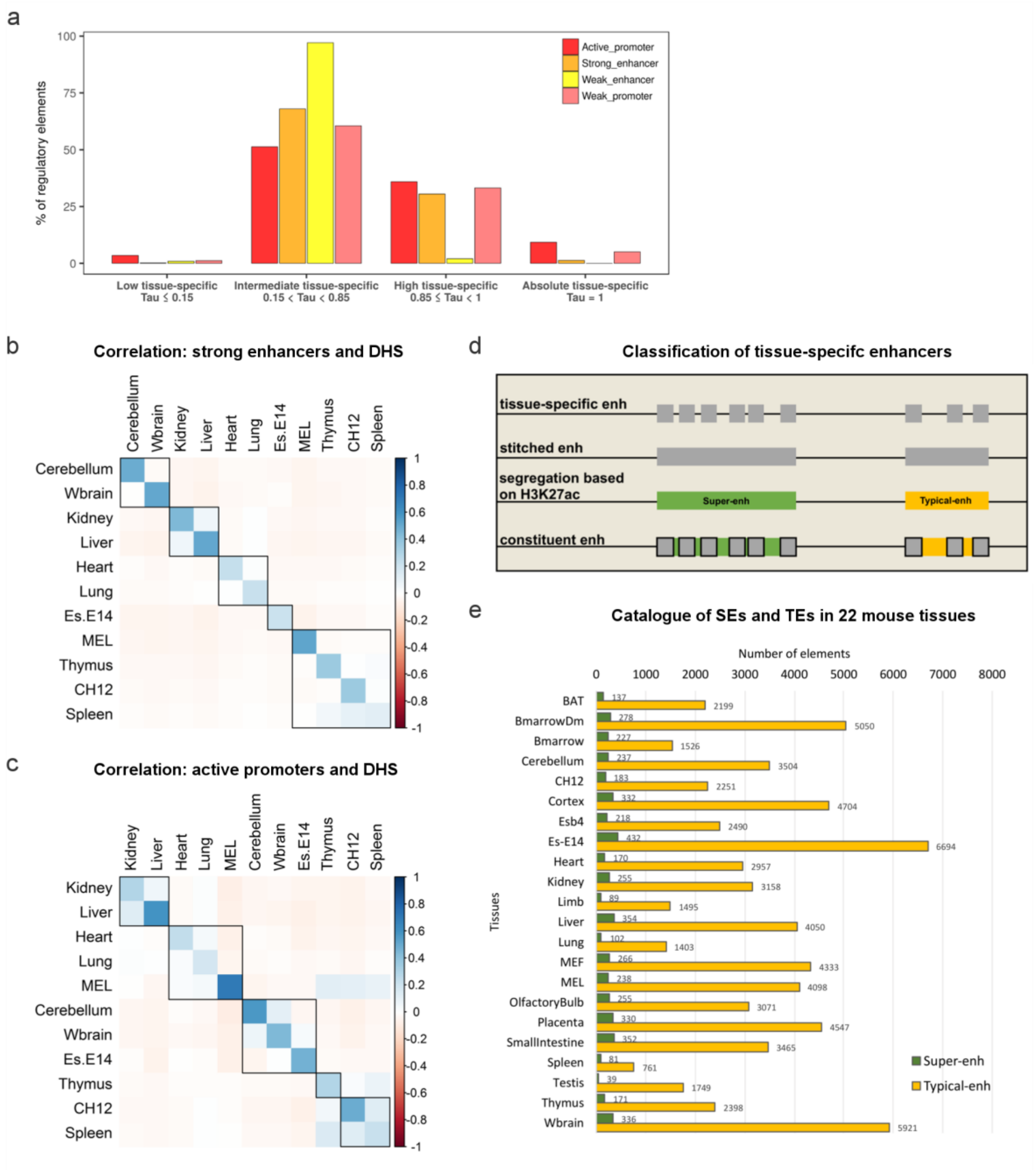

Figure S2: Overview of tissue-specific regulatory elements in the mouse genome. (a) Percentage of regulatory elements across chromatin states categorised according to their tissue-specificity index (measured by Tau_{reg}). (b-c) Heatmaps showing pairwise Pearson's correlations between tissue-specific strong enhancers and DHS (b); and tissue-specific active promoters and DHS (c). The order of the tissues is sorted by hierarchical clustering and the boxes represents clusters obtained from the clustering of the correlation matrix. (d) A schematic illustrating the methodology used to classify tissue-specific enhancers into super-enhancers (SEs) and typical-enhancers (TEs). The tissue-specific enhancers are stitched together into cohesive units and ranked on their H3K27ac occupancy. High ranking stitched enhancers are defined as SEs. The original tissue-specific enhancer elements within SEs and TEs are referred to as constituent enhancers. (e) Bar plot displaying the number of SE and TE elements identified in each tissue.

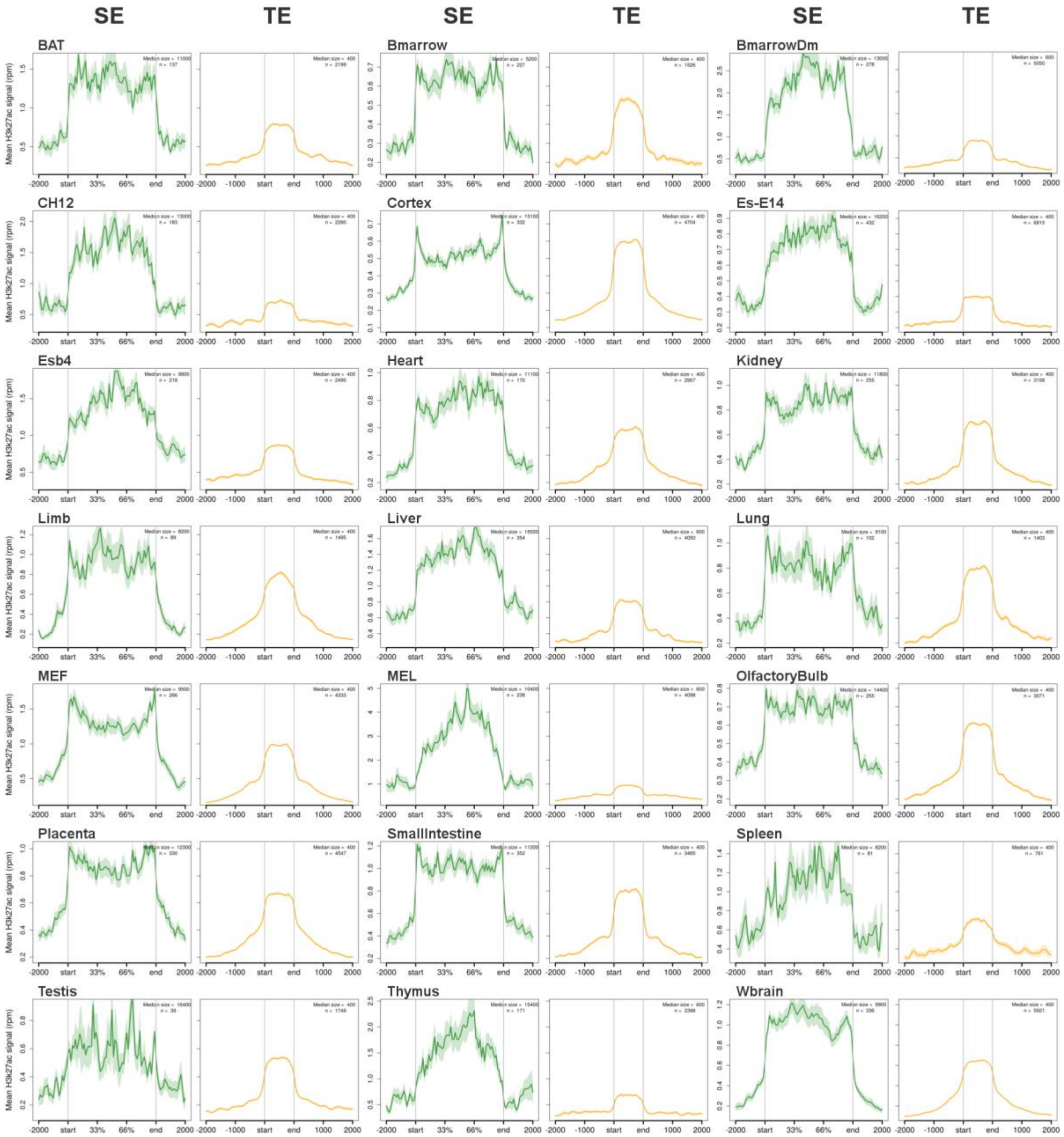

Figure S3: H3K27ac activity within SEs and TEs. Metagene profiles displaying mean H3K27ac ChIP-seq signal across all the SE and TE regions in each tissue. The profiles are centred on the enhancer region and the width represents the median length of the enhancers. An additional 2 kb region flanking each enhancer element is also shown.

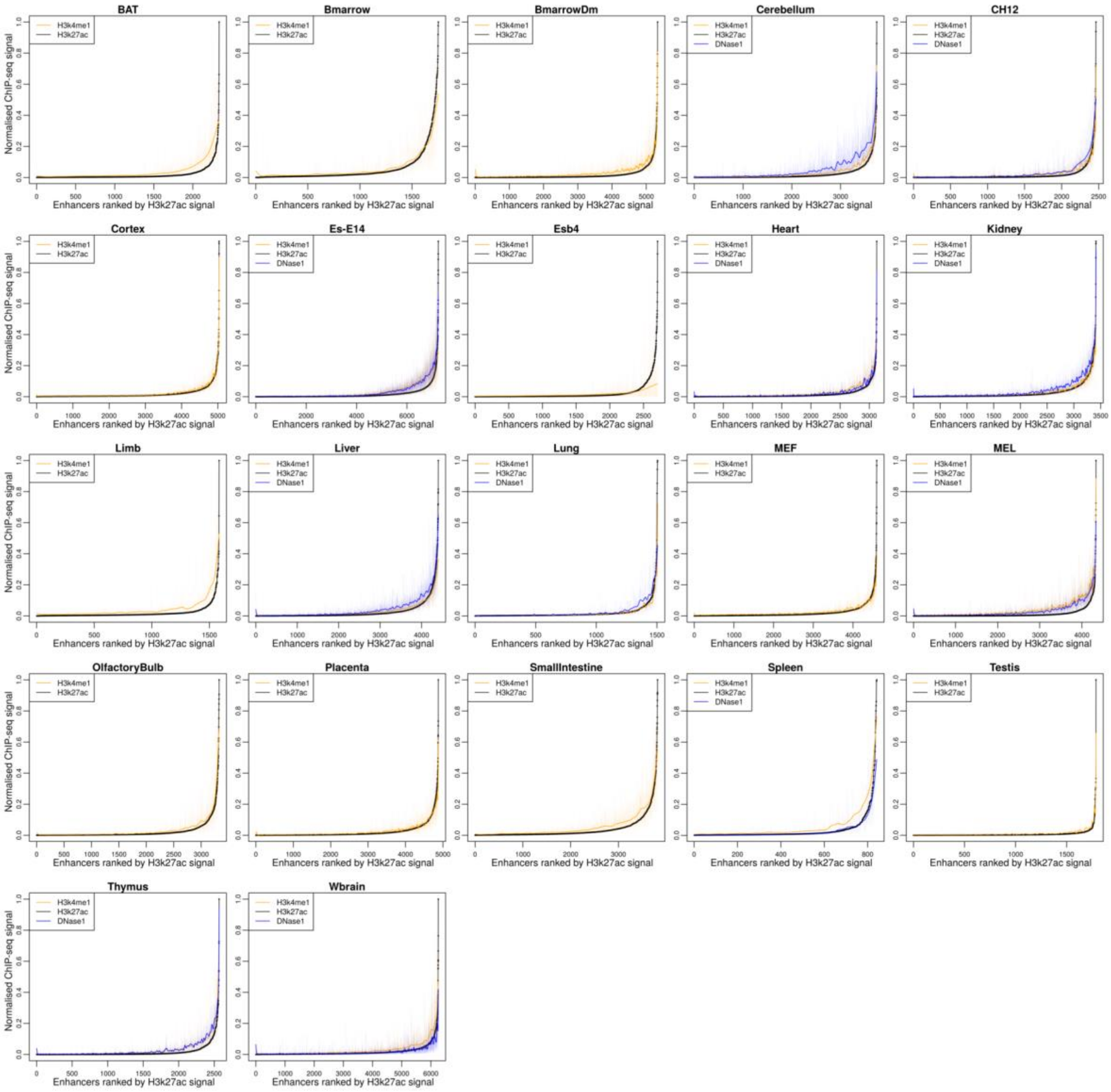

Figure S4: Enrichment of chromatin marks over stitched cohesive enhancer units. Distribution of H3K4me1, H3K27ac and DNase1 signal across stitched tissue-specific enhancers. The plot was normalised by dividing the input-subtracted ChIP-seq signal for each enhancer by the maximum ChIP-seq signal detected in each feature. The stitched enhancers for each feature on x-axis are ranked according to the H3K27ac ChIP-seq signal. Note: DNase-seq data was not available for all tissues.

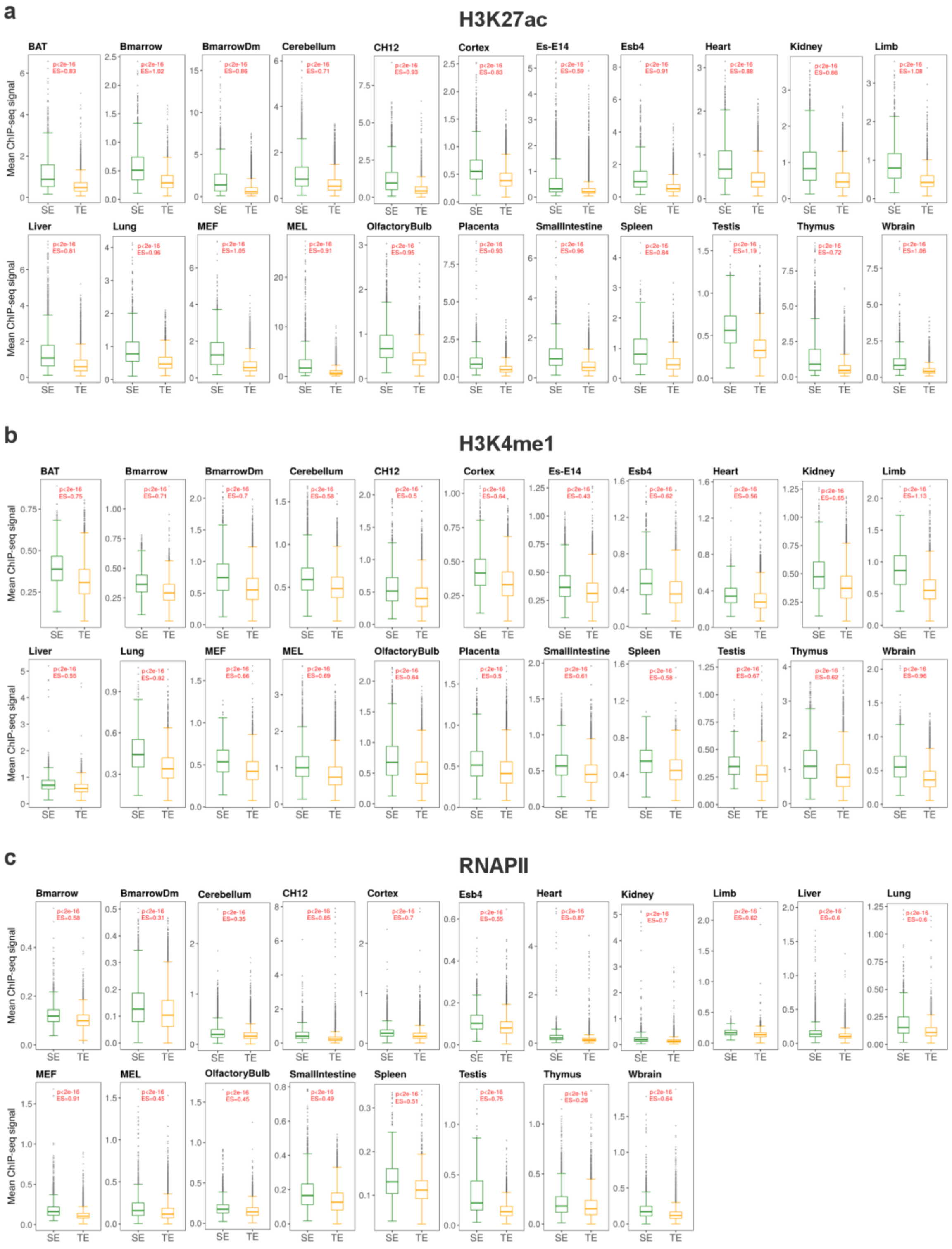

Figure S5: Chromatin activity within SE and TE constituent enhancers: Comparison of H3K27ac (a), H3K4me1 (b) and RNA polymerase II (c) ChIP-seq signal between constituent enhancers within SEs and TEs in every tissue. p: p-values from Wilcoxon Rank Sum Test; ES: effect size.

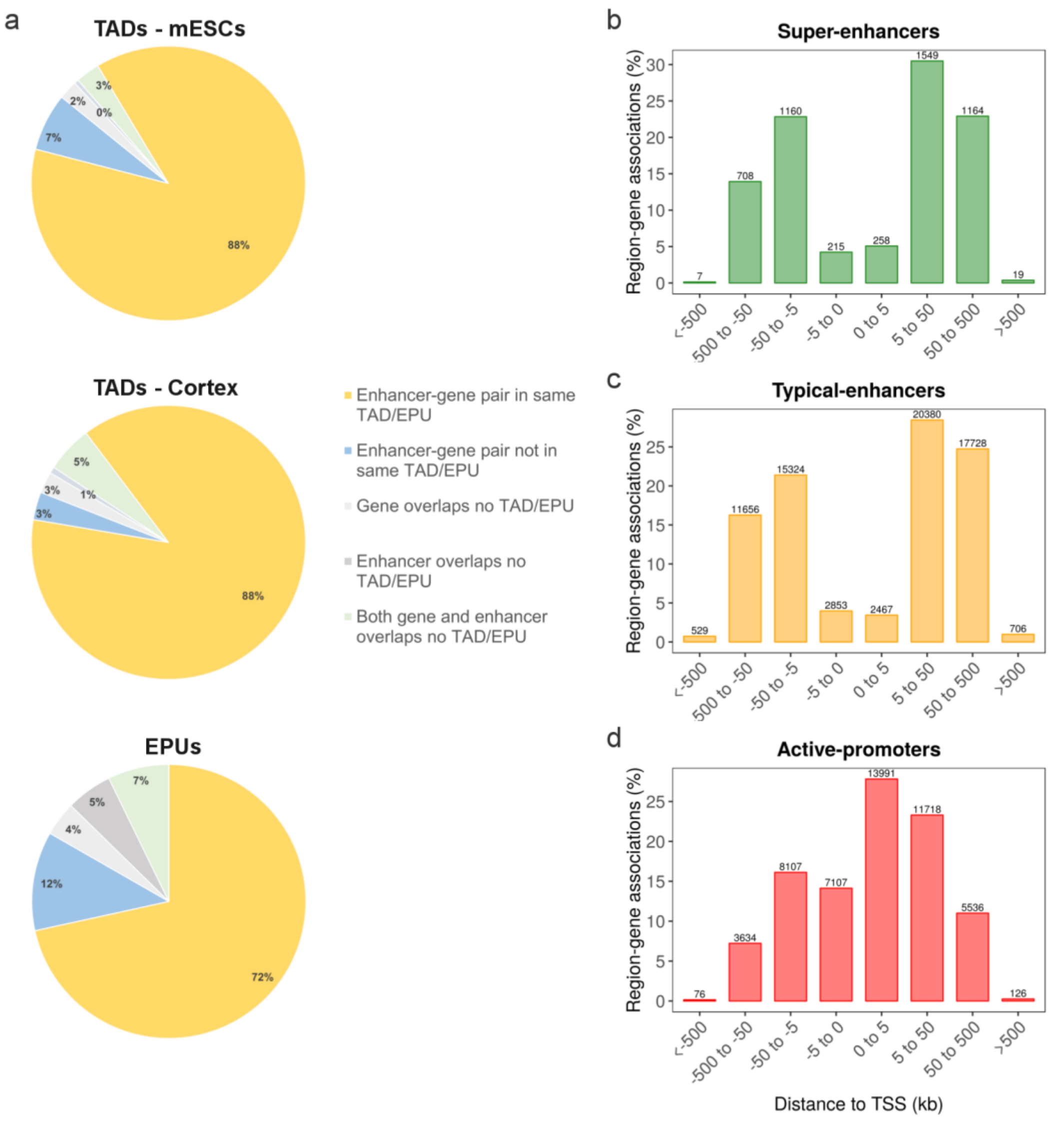

Figure S6: Region-gene associations of regulatory elements. (a) Pie charts displaying the proportion of gene-enhancer pairs within previously reported TADs and EPUs in the mouse genome. (b-d) Bar plots binned by orientation and TSS showing the distance between various regulatory elements and their putative target genes. For all three graphs, the y-axis represents the percentage of region-gene associations while the number of associations in each bin are listed in the graph. The x-axis shows the distance (divided into separate bins) of the region relative to the TSS of gene. Negative distance depicts regions upstream of TSS; positive distance depicts regions downstream of TSS; 0 represents the TSS.

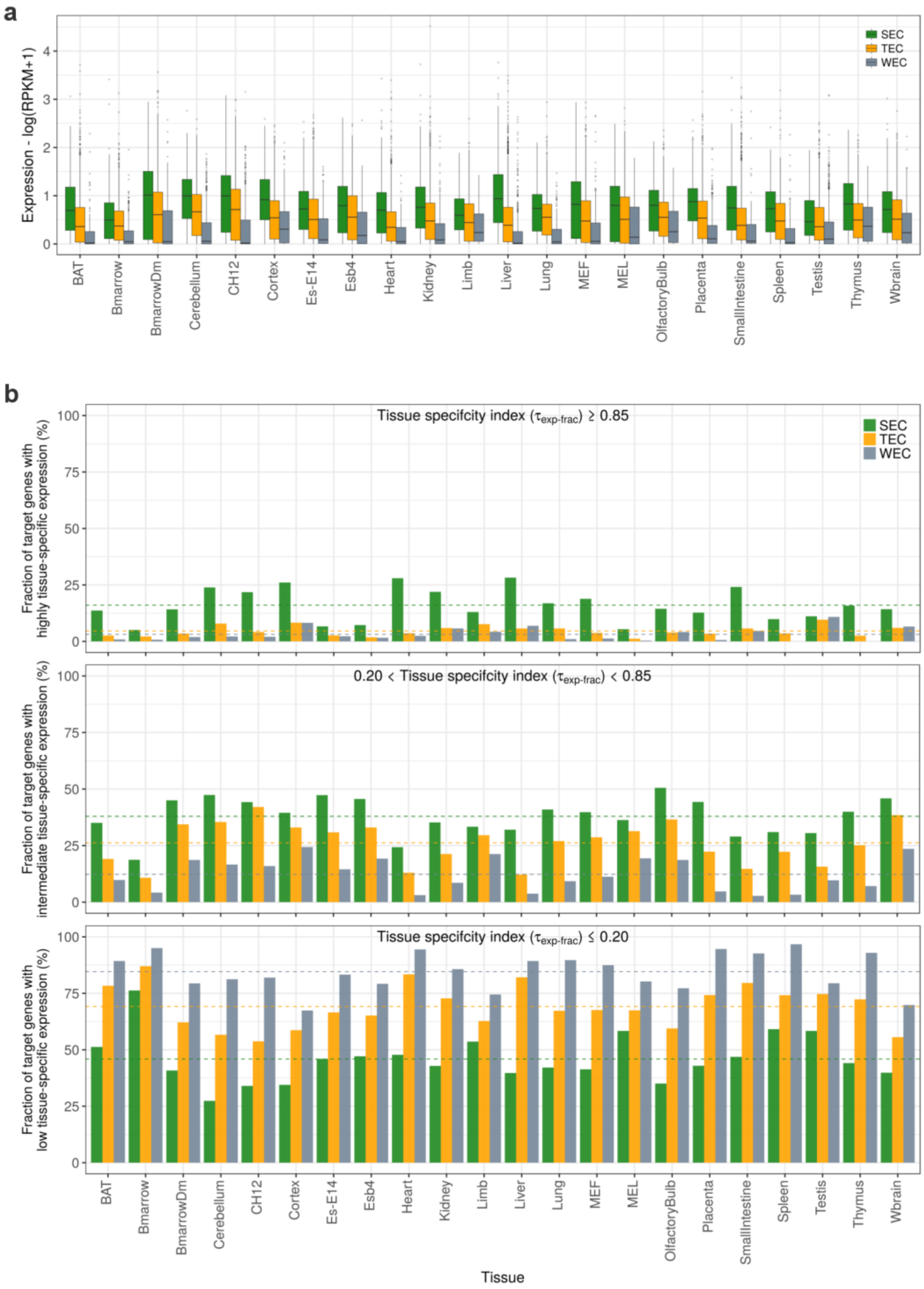

Figure S7: Relationship between enhancer activity and their target gene expression. (a) Box plot displaying the total-expression of genes in each enhancer class. (b) Bar plots showing the proportion of genes with high tissue-specific expression ($Tau_{exp\text{-}frac} \geq 0.85$), intermediate tissue-specific expression ($0.20 < Tau_{exp\text{-}frac} < 0.85$) and low tissue-specific expression ($Tau_{exp\text{-}frac} \leq 0.20$) in each enhancer class. The dotted lines show the mean across all the tissues. SEC: super-enhancer class; TEC: typical-enhancer class; WEC: weak-enhancer class.

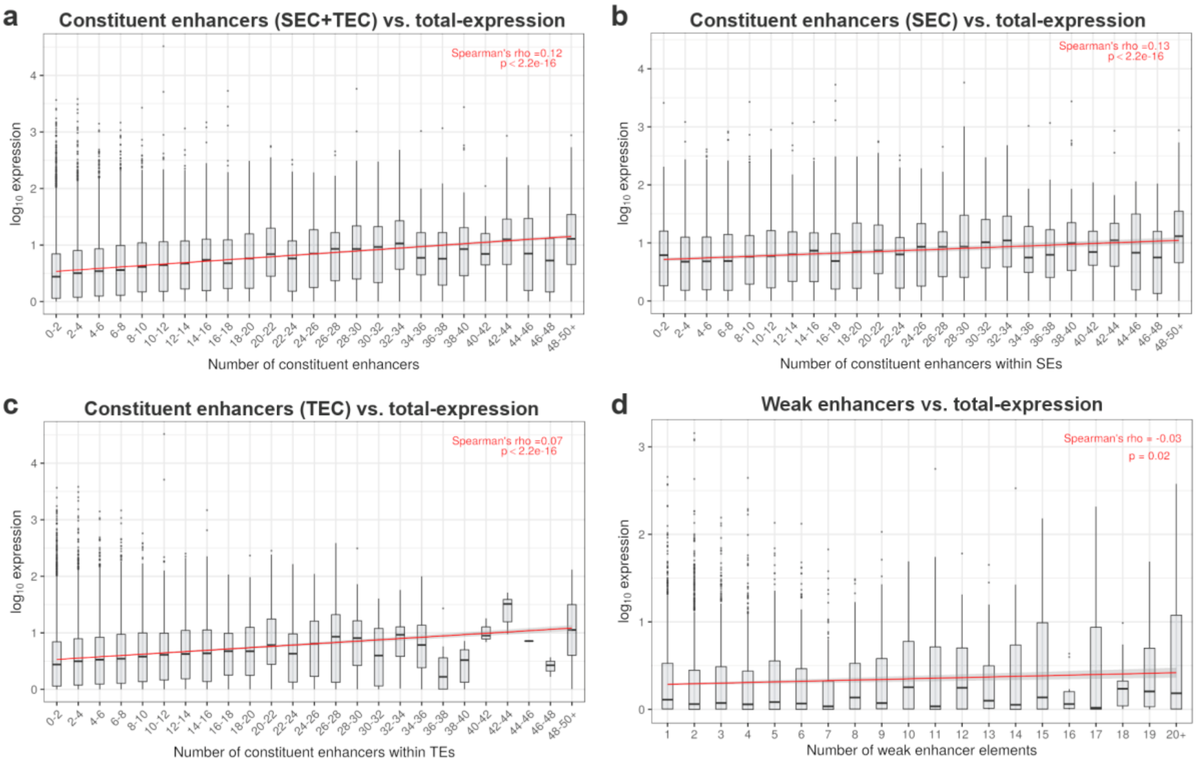

Figure S8: Impact of constituent enhancer density on target gene expression. (a) Correlation between number of constituent enhancers (within SEs and TEs combined) and total-expression of their associated genes. (b) Correlation between number of constituent enhancers within SEs and total-expression of their associated genes. (c) Correlation between number of constituent enhancers within TEs and total-expression of their associated genes. (d) Correlation between number of weak enhancers elements and total-expression of their associated genes. The trend lines were calculated using linear regression.

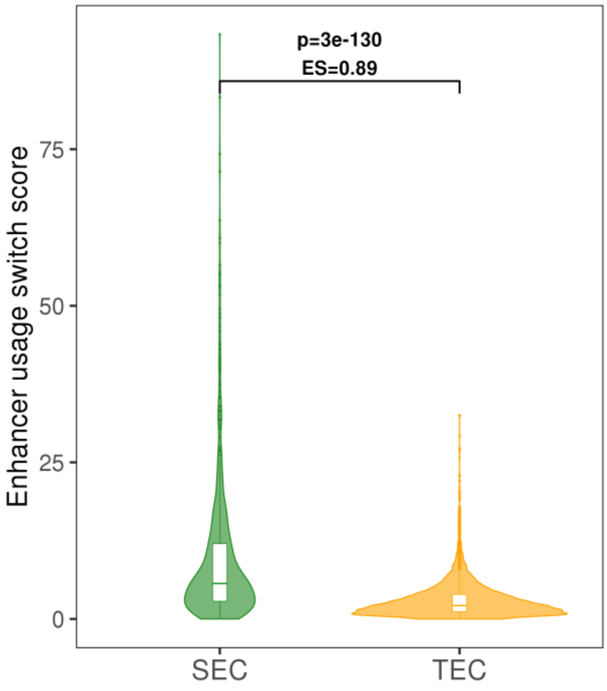

Figure S9: Enhancer usage switch associated with genes within SEC and TEC with multiple enhancer tissue-types. Violin plots displaying the enhancer usage switch scores associated with the genes within SEC and TEC. The p-values were computed using the Wilcoxon Rank Sum Test. p: p-value; ES: effect size.

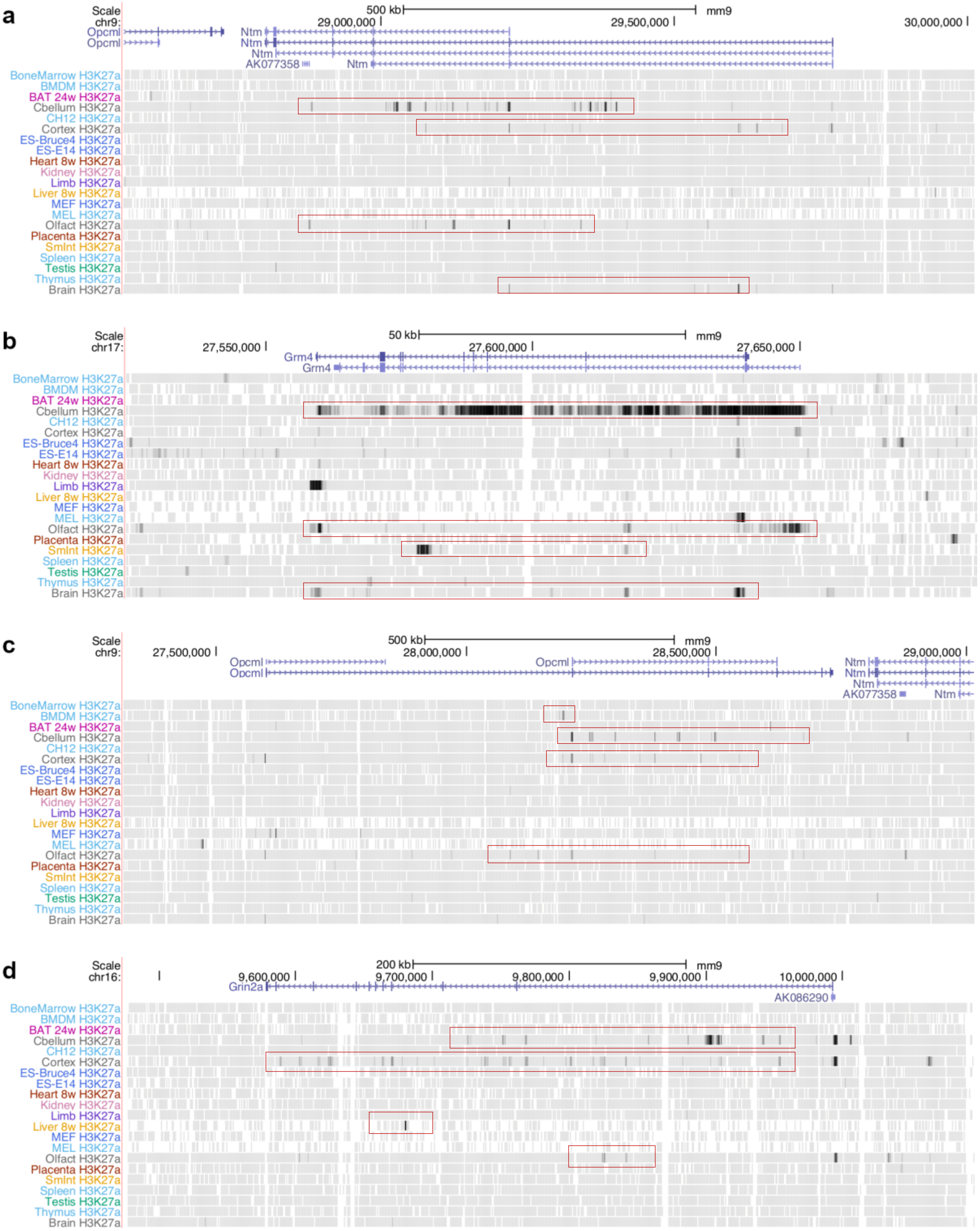

Figure S10: Genomic view of genes demonstrating enhancer usage switch. Genome browser snapshots of genes associated with SEs (panel a and b) and TEs (panel c and d), displaying H3K27ac ChIP-seq signal (from ENCODE) across a wide range of tissues. Each track shows grey boxes where the degree of darkness corresponds to H3K27ac enrichment signal (darker grey = higher enrichment). The red boxes highlight active regulatory regions as indicated by high H3K27ac signal. The number of active regulatory regions associated with the genes differ across the tissues, indicating their enhancer usage vary across different tissues.

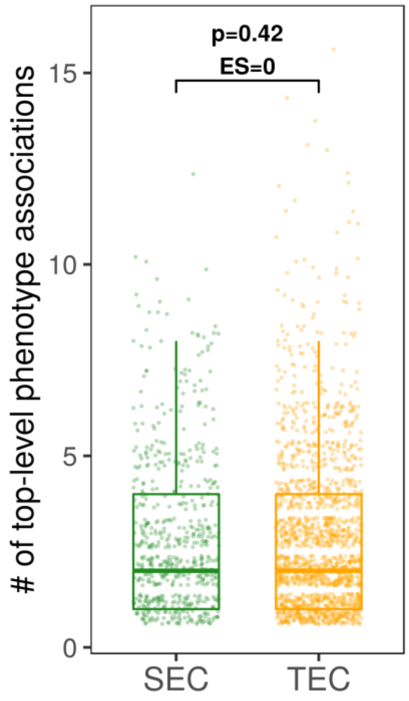

Figure S11: Breadth of phenotypes associated with SE and TE gene knockouts in mouse. Box plot displaying the number of top-level phenotype terms associated with SE and TE gene knockouts in the IMPC. The p-values were computed using the Wilcoxon Rank Sum Test. p: p-value; ES: effect size.

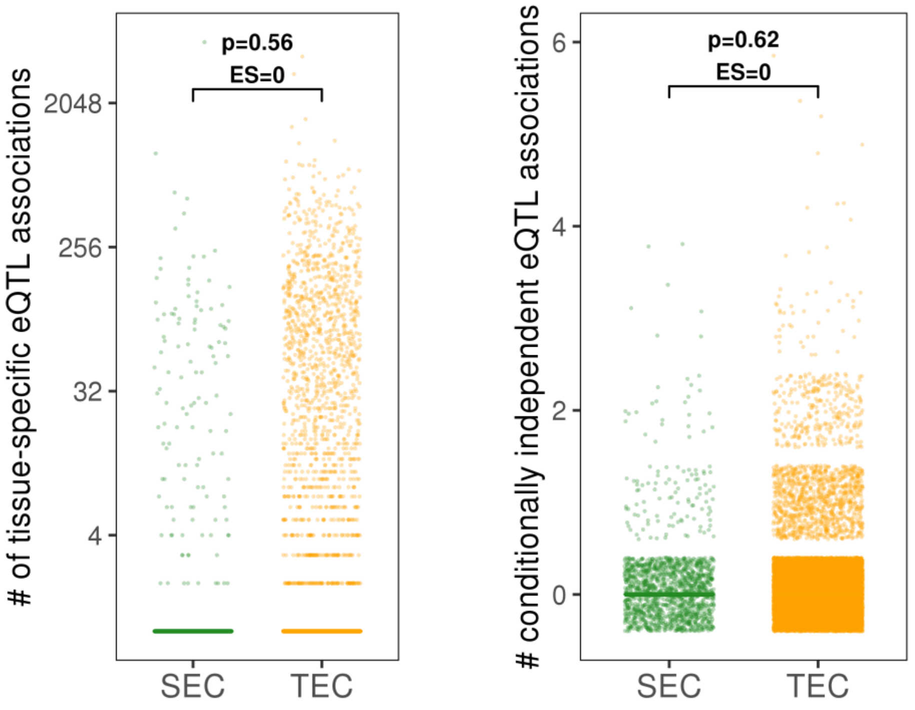

Figure S12: Number of eQTLs associated with genes within SEC and TEC. Box plots displaying the total count of significant eQTLs associated with each gene within SEC and TEC. Plot on the left displays the number of eQTL associations calculated using the significant variant-gene association dataset based on permutations in each tissue from GTEx. Plot on the right displays the number of eQTL associations calculated using the conditionally independent eQTL dataset mapped using stepwise regression. The p-values were computed using the Wilcoxon Rank Sum Test. p: p-value; ES: effect size.

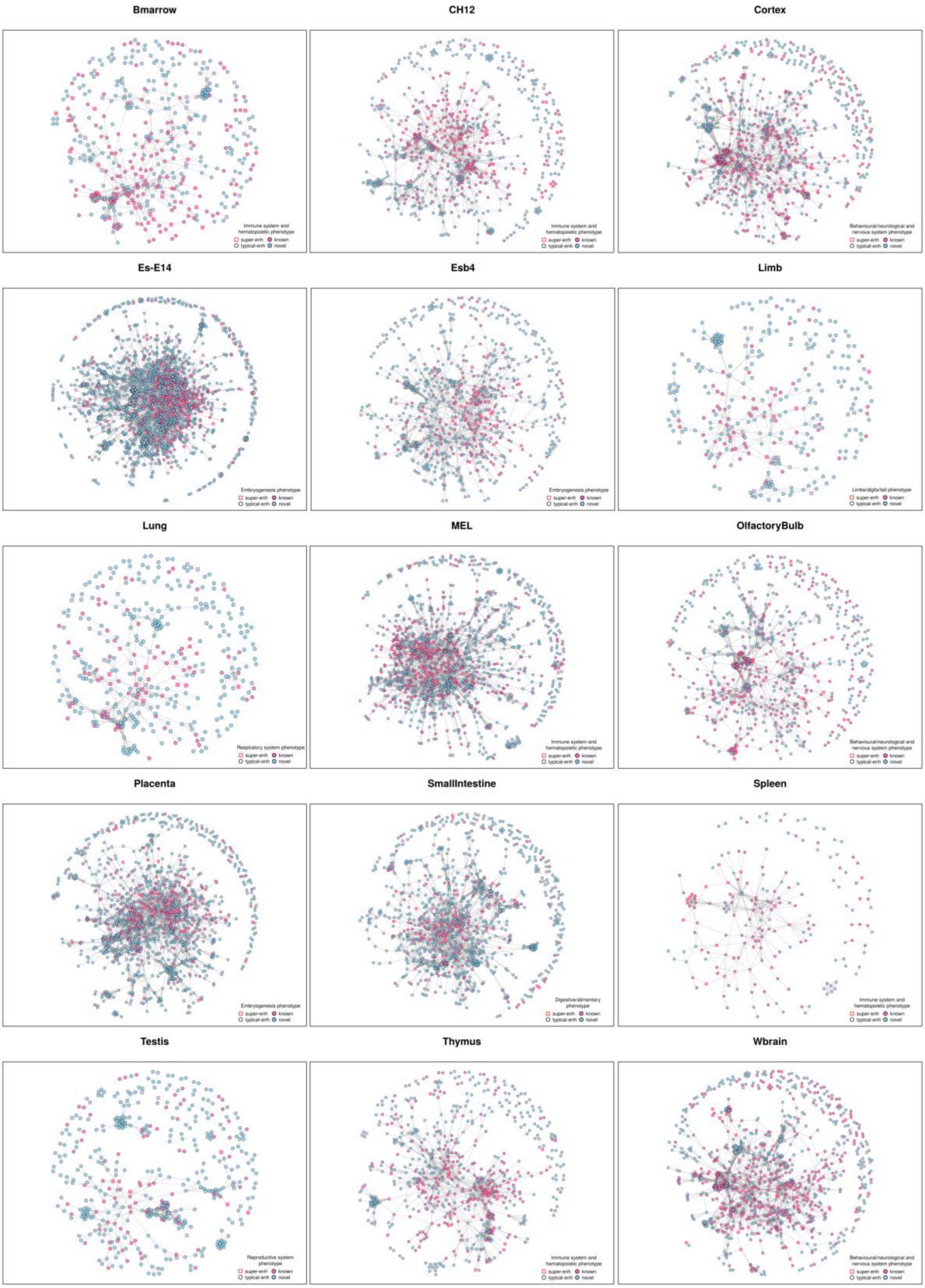

Figure S13: Protein-protein interaction maps of enhancer associated genes. Nodes in each network represents enhancer associated genes and edges represent a potential protein-protein interaction between them. Genes associated with tissue-type relevant phenotypes are highlighted in pink and the shape of the node displays SE and TE associated genes.

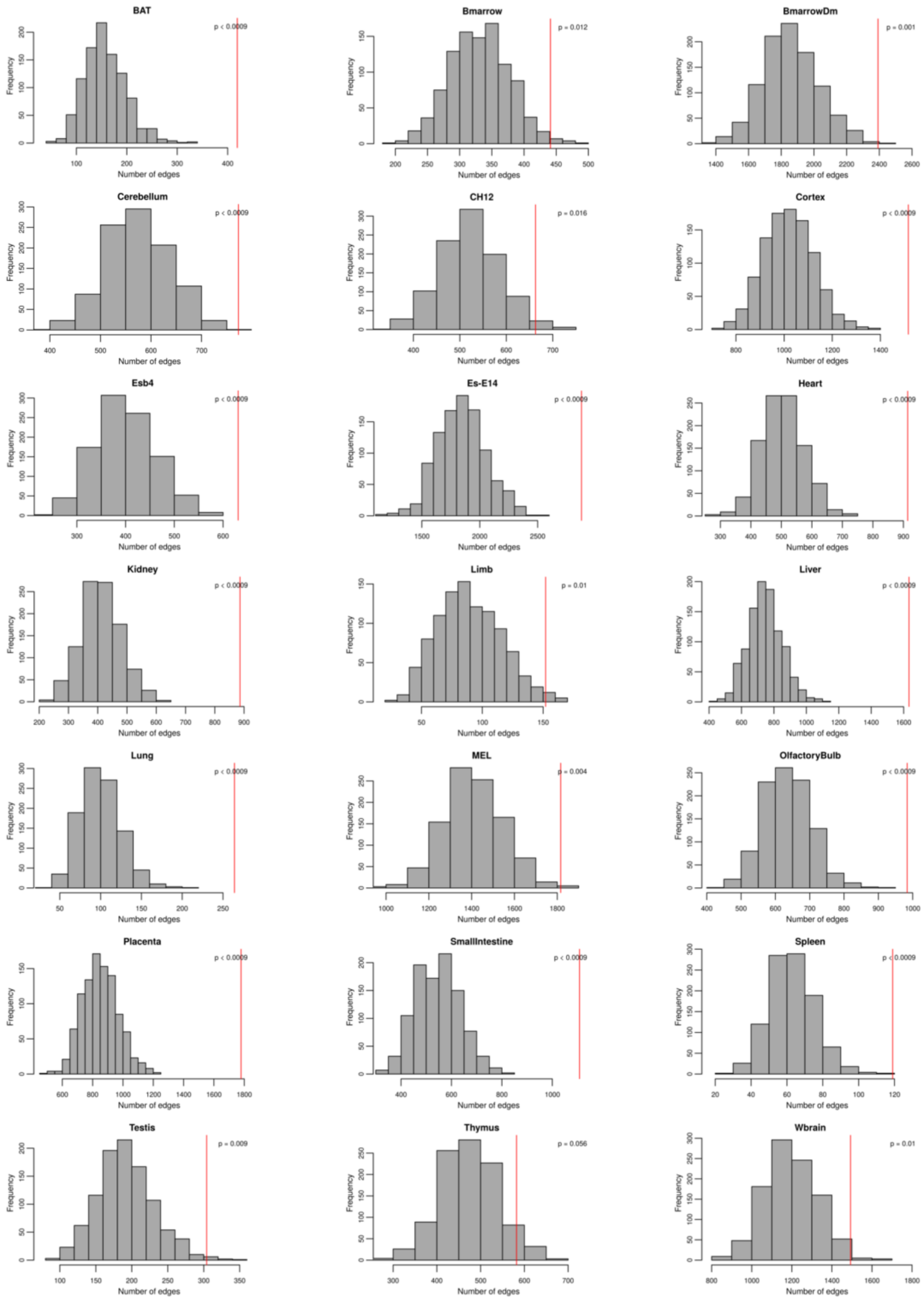

Figure S14: Protein-protein interaction simulations. Histograms showing the protein-protein interaction simulations performed for every tissue. The PPI networks were simulated 1000 times by adding randomly selected protein-coding genes (equal to the number of phenotype associated genes in each tissue) to the network and counting their edges with novel genes. The red vertical line shows the observed number of edges between novel and phenotype associated genes and the grey distribution (obtained from permutation) shows the number of edges between novel and randomly added genes. The p-value shown was calculated by dividing the number of permuted values larger than the observed value by the total number of items in the permutation distribution (i.e. 1001).

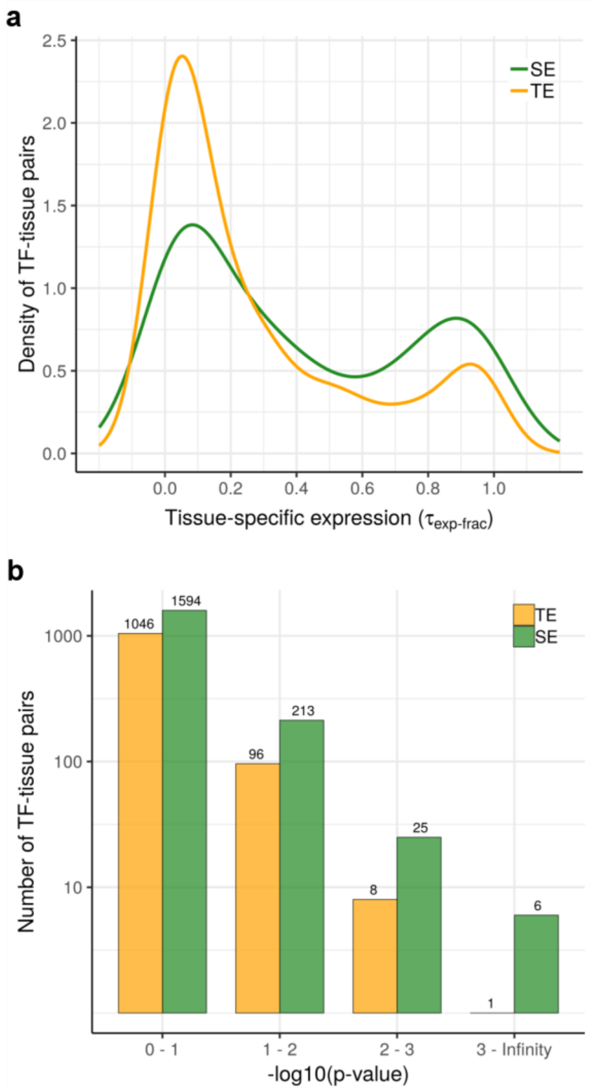

Figure S15: Transcription factor binding within SE and TE constituents. (a) Density plot showing the distribution of TFs whose cistrome significantly colocalise with enhancer segments, plotted against the tissue-specific expression of the TF in the corresponding tissues. (b) Bar plot displaying the number of TF-tissue pairs which have significantly greater TFBS density in SE compared to TEs (green bars), and vice-versa for TEs (orange bars). For each TF-tissue pair, a Wilcoxon Rank Sum Test was used to compare its TFBS density between SEs and TEs. The TF-tissue pairs are binned by the logarithmic significance of the difference in TFBS density between SEs and TEs obtained from the Wilcoxon Rank Sum Test. For e.g. the first pair of bars represents the TF-tissue pairs with a p-value of difference lying in range $0 \leq -\log10(\text{p-value}) < 1$.

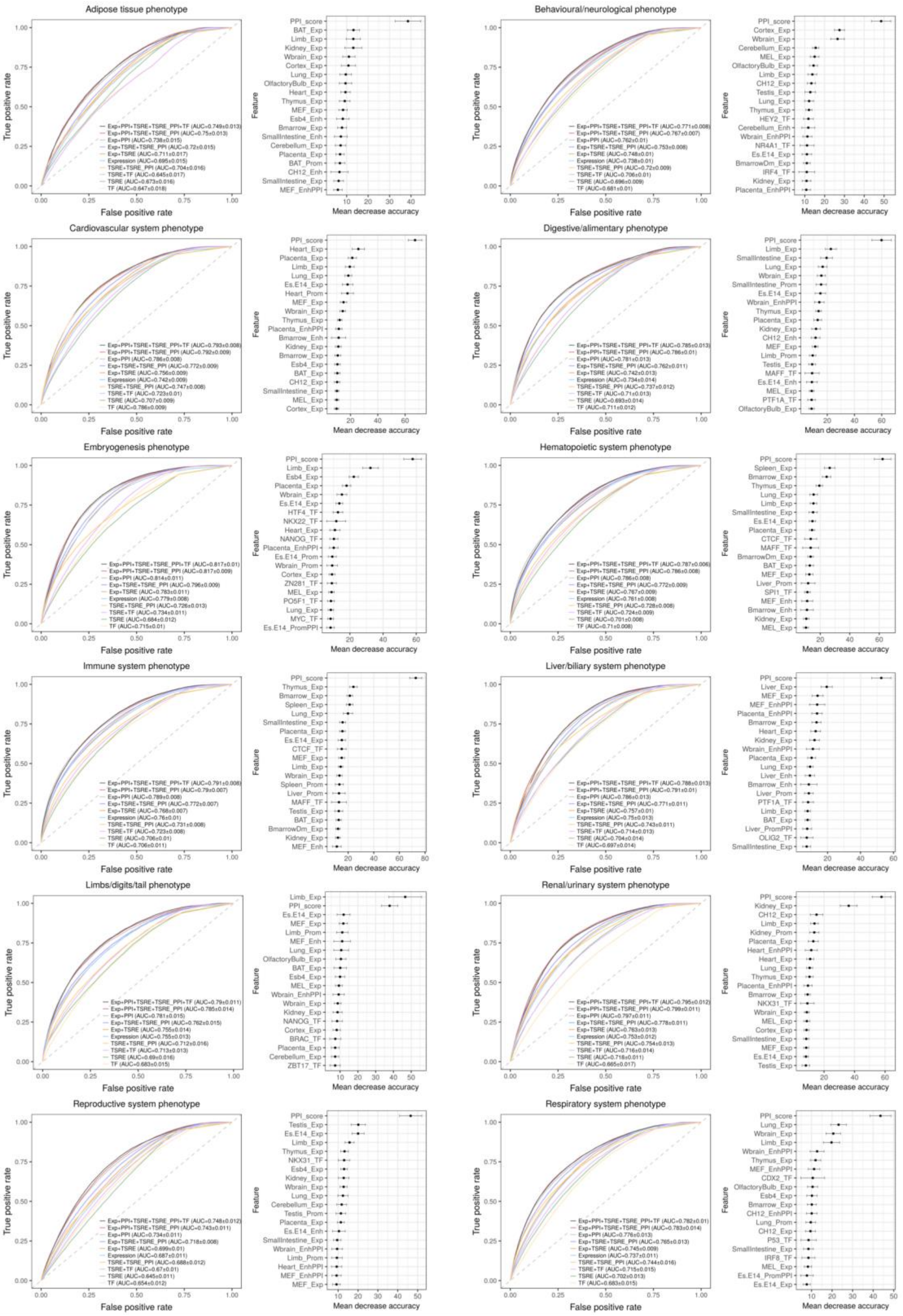

Figure S16: Performance of random forest classifiers to predict mammalian gene-phenotype associations. Receiver operating characteristic (ROC) curves comparing the performance of 10 random forest classifier models used to predict gene-phenotype associations in various phenotypic domains. Several random forest classifiers were modelled for every phenotype using protein-protein interaction data (PPI), expression (Exp), transcription factor(TF) and tissue-specific regulatory element (TSRE) data. On the right is shown the feature importance chart of the best performing model (Exp+PPI+TSRE+TSRE_PPI+TF) showing the top 20 predictor variables measured by the mean decrease in accuracy. Error bars display the standard deviation.

a

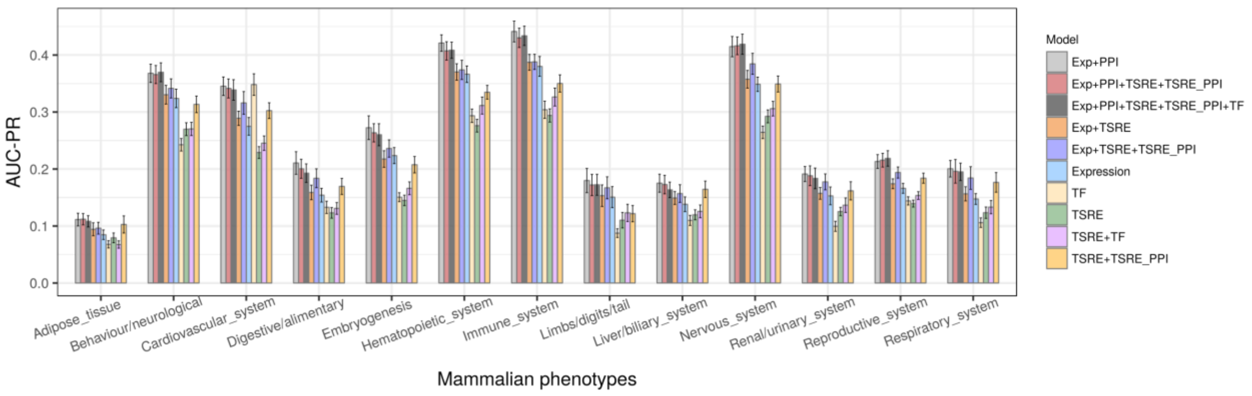

b

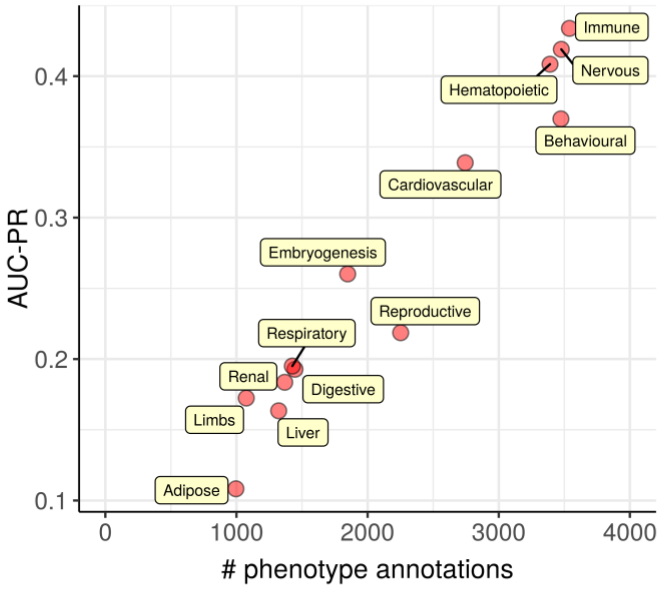

Figure S17: Precision and recall of classifiers used to predict gene-phenotype associations. (a) Bar plot comparing the predictive power of various random forest models to infer different phenotypes, based on 5-fold cross validation repeated 10 times. The bars represent the mean area under the curve measured for precision-recall (AUC-PR) and the error bars display the standard deviation. The range of y-axis was adjusted to clearly show the differences between the models. (b) Scatter plot showing the relationship between the precision rate and number of known phenotype annotation counts in MGD for each phenotype domain.

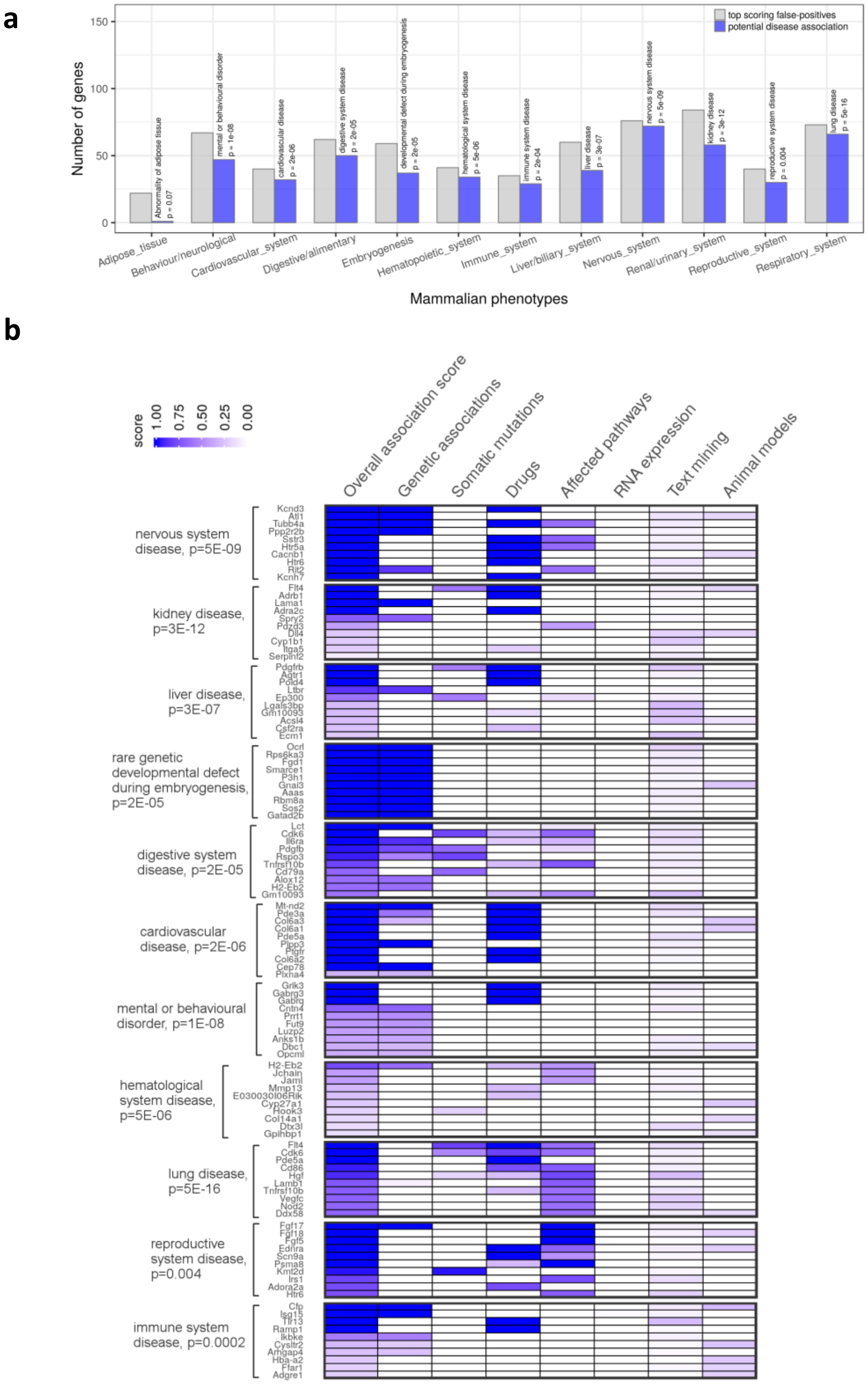

Figure S18: Evaluation of top-scoring false-positives using the Open Targets platform. (a) Bar plot displaying the number of genes in each phenotype domain with potential association to the corresponding disease. (b) Heatmap displaying the top 10 gene predictions in different phenotype domains along with the evidence source for their potential association to the corresponding disease. The Open Targets Platform was used to validate the novel predictions and link them to potential diseases using integrated genome-wide public datasets.

Table S1: Mammalian phenotype and human disease ontology terms enriched in genes associated with weak-enhancers. Listed are the most enriched mammalian phenotypes and human disease ontology terms in each tissue. The associated FDR values are reported next to the enriched terms and was calculated using Benjamini-Hochberg method. *N* displays the number of weak-enhancer associated genes in each group.

Weak-enhancers

	N	Mouse Phenotypes	Disease
BAT	214	○ -	○ Otofaciocervical Syndrome, 10^{-3}
BmarrowDm	939	○ -	○ Autism Spectrum Disorders, 10^{-3}<br>○ Mental Retardation, X-Linked, 10^{-3}
Bmarrow	368	○ -	○ -
Cerebellum	1202	○ abnormal innervation, 10^{-5}<br>○ abnormal synaptic transmission, 10^{-4}	○ Autistic Disorder, 10-^{10}<br>○ Schizophrenia,10-^{6}
CH12	1117	○ abnormal cartilage morphology, 10^{-2}<br>○ perinatal lethality, 10^{-2}	○ Mental Depression, 10^{-4}<br>○ Alcoholic Intoxication, Chronic, 10^{-4}
Cortex	1006	○ abnormal synaptic transmission, 10^{-14}<br>○ abnormal nervous system physiology, 10^{-13}	○ Bipolar Depression, 10^{-12}<br>○ Schizophrenia, 10^{-11}
Esb4	391	○ increased neurotransmitter release, 10^{-3}	○ Autistic Disorder, 10^{-2}
Es-E14	687	○ perinatal lethality, 10^{-6}<br>○ neonatal lethality, 10^{-5}	○ Autism Spectrum Disorders, 10^{-3}<br>○ Craniofacial Abnormalities, 10^{-2}
Heart	335	○ abnormal muscle contractility, 10^{-4}<br>○ increased heart ventricle size, 10^{-3}	○ -
Kidney	449	○ -	○ Alcoholic Intoxication, Chronic, 10^{-2}<br>○ Autism Spectrum Disorders, 10^{-2}
Limb	87	○ abnormal palatal shelf fusion at midline, 10^{-3}<br>○ cleft hard palate, 10^{-2}	○ -
Liver	922	○ -	○ Attention deficit hyperactivity, 10^{-5}<br>○ Autistic Disorder, 10^{-5}
Lung	286	○ abnormal olfactory lobe morphology, 10^{-3}<br>○ abnormal nervous system physiology, 10^{-2}	○ -
MEF	601	○ muscle phenotype, 10^{-3}<br>○ abnormal limb bone morphology, 10^{-3}	○ Ventricular Septal Defects, 10^{-2}<br>○ Alcoholic Intoxication, Chronic, 10^{-2}
MEL	621	○ -	○ Autistic Disorder, 10^{-4}<br>○ Mental Retardation, X-Linked, 10^{-4}
OlfactoryBulb	360	○ abnormal motor coordination/balance, 10^{-4}<br>○ abnormal nervous system physiology, 10^{-3}	○ Autistic Disorder, 10^{-4}<br>○ Fragile X Syndrome, 10^{-3}
Placenta	975	○ abnormal neuron differentiation, 10^{-3}<br>○ abnormal respiratory system physiology, 10^{-3}	○ Central neuroblastoma, 10^{-7}<br>○ Alzheimer's Disease, 10^{-7}
SmallIntestine	310	○ abnormal gallbladder physiology, 10^{-3}<br>○ postnatal lethality, 10^{-2}	○ Obesity, 10^{-2}
Spleen	170	○ abnormal semicircular canal morphology, 10^{-3}<br>○ abnormal otolith organ morphology, 10^{-2}	○ Hypoplastic cochlea, 10^{-2}<br>○ Congenital Abnormality, 10^{-2}
Testis	864	○ abnormal neuron morphology, 10^{-3}<br>○ abnormal synaptic transmission, 10^{-3}	○ Autistic Disorder, 10^{-4}<br>○ Craniofacial Abnormalities, 10^{-3}
Thymus	412	○ -	○ -
Wbrain	776	○ abnormal neuron differentiation, 10^{-10}<br>○ abnormal nervous system development, 10^{-8}	○ Schizophrenia, 10^{-6}<br>○ Autism Spectrum Disorders, 10^{-6}
